# Grain Boundary Segregation Suppresses Local Short‐Range Ordering in Nanocrystalline High‐Entropy Alloys

**DOI:** 10.1002/advs.202515510

**Published:** 2025-11-30

**Authors:** Moses A. Adaan‐Nyiak, Mack Cleveland, Benjamin Hewitt, Ericmoore Jossou, Mehmet Topsakal, Simerjeet K. Gill, Sooyeon Hwang, Kim Kisslinger, Ahmed A. Tiamiyu

**Affiliations:** ^1^ Department of Mechanical and Manufacturing Engineering University of Calgary 2500 University Drive NW Calgary Alberta T2N 1N4 Canada; ^2^ Department of Materials Science and Engineering Massachusetts Institute of Technology Cambridge MA 02139 USA; ^3^ Department of Nuclear Science and Engineering Massachusetts Institute of Technology Cambridge MA 02139 USA; ^4^ Department of Electrical Engineering and Computer Science Massachusetts Institute of Technology Cambridge MA 02139 USA; ^5^ Nuclear Science and Technology Department Brookhaven National Laboratory Upton NY 11973 USA; ^6^ Center for Functional Nanomaterials Brookhaven National Laboratory Upton NY 11973 USA

**Keywords:** grain boundary segregation, multicomponent alloys, nanocrystalline materials, short‐range ordering, Warren‐Cowley coefficient

## Abstract

Multi‐principal‐element alloys like high‐entropy alloys (HEAs) have potential applications in many engineering fields due to their unique mechanical/functional properties. While HEAs are generally considered random solid solutions, recent studies revealed that they are prone to short‐range‐ordering (SRO) due to the complex multi‐pair‐wise interactions among the constituent elements. Meanwhile, SROs' evolution can sometimes be deleterious, and it is necessary to have control over their evolution. Examining the AlCoCrFe‐Zr model alloy, long‐range ordering occurs following the expectation of enthalpic predictions. Advanced characterization techniques—transmission electron microscopy, high‐energy synchrotron X‐ray diffraction/pair distribution function, and atom probe tomography, reveal that SRO is suppressed in as‐milled and GB‐decorated NC‐(AlCoCrFe)100‐xZrx (x = 0–1.5 atomic %). Warren‐Cowley coefficient calculations are further used to validate the suppression of SRO. Besides the low segregation enthalpies of Cr, Fe, and Zr, and the high‐mixing enthalpy of Cr and Fe, the short diffusion path to GBs due to high‐GB density in the NC‐HEAs and the higher energy state of the GBs than the matrix promotes GB‐segregation that further alters the matrix chemistry and consequently disfavors SRO formation within the matrix. Despite the GB‐segregation of Cr, Fe, and Zr, the matrices and GBs remain in a random solid solution.

## Graphical abstract

Multicomponent alloys, such as high‐entropy alloys (HEAs), are considered to be in a random solid solution. However, recent studies have shown that short‐range ordering (SRO) is prevalent in these HEAs due to multipairwise interactions among the constituent elements, which can be deleterious. This research shows that grain boundary segregation suppresses SRO in nanocrystalline multicomponent alloys.

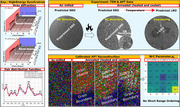

## 1 Introduction

Understanding the local structures or atomic displacements in structural materials is essential to assessing the origin of the unique properties they exhibit.^[^ ^1^, ^2^ ^]^ This local structural information correlates strongly with the *local chemical ordering*, which results from the arrangement of atoms in neighboring atomic shells and within local domains in solid solutions. While a more negative mixing enthalpy favors local chemical ordering,^[^ ^3^ ^]^ they can manifest in several ways including sub‐nanoscale *short‐range ordering* (SRO) structures whose size is usually < 1 nm (and it spans over mainly the nearest and second nearest neighbor shells), nanoscale *medium‐range ordering* structures whose size is in the range of 1‐5 nm, and *long‐range ordering* (LRO) nanodomains (usually nanoprecipitates) with dimensions > 5 nm.^[^ ^3^, ^4^ ^]^ The phenomenon of SRO, sometimes called *chemical short‐range ordering* or *local short‐range ordering*, has been reported in traditional alloy systems, and it is found to significantly influence their structural stability, magnetic, and mechanical properties.^[^ ^5^ ^]^ For instance, there is an increase in strength in Ni‐Mo alloy^[^ ^6^ ^]^ and high‐manganese austenitic steel^[^ ^5^ ^]^ due to SRO; SRO impeded dislocation motion and hence improved yield strength.

In multi‐principal‐element alloys such as medium‐ to high‐entropy alloys (HEAs), a complex local structure is typically expected to play a significant role in their unusual mechanical and physical properties. HEAs are a new class of alloys that comprise four or more principal elements in equiatomic or near equiatomic ratios, with atomic concentration in the range of 5–35 atomic % (at. %) that form solid solutions with simple crystal structures due to the associated high configuration entropy.^[^ ^7^ ^]^ HEAs have a plethora of intriguing properties, including, but not limited to, high strength and hardness, improved corrosion resistance, excellent oxidation and wear resistance, improved ductility, and thermal stability. These properties are reportedly derived from their unique core effects,^[^ ^8^, ^9^ ^]^ including the SRO that results from the preferential clustering of specific constituent elements in local lattices.^[^ ^10^ ^]^ Recent studies^[^ ^11^, ^12^, ^13^ ^]^ have revealed that the distribution of atoms in HEAs is not entirely in an ideal disordered state, but rather, there is a preference for complex pairwise interactions.^[^ ^1^ ^]^ The formation of SRO has since been commonly known to be an important structural feature in HEAs.^[^ ^14^ ^]^

Zhao^[^ ^10^ ^]^ investigated the role of SRO on defect diffusion and migration in CuNiCoFe HEA using three different models: the average atom model, the random model, and a model with SRO. Significant suppression of defect cluster motion is observed in the model with SRO compared to the average atom and random models; the SROs serve as cluster pinning points. Using atomic‐scale simulations and synchrotron X‐ray scattering to quantify the degree of chemical SRO and to perform small‐angle X‐ray scattering, Wang and co‐workers^[^ ^14^ ^]^ reported that the presence of SRO in a BCC TiZrHfNb refractory HEA improved strength, elastic modulus, and hardness. Ding et al. ^[^ ^15^ ^]^ show that SRO correlates with stacking fault energy, which enhances strength, ductility, and toughness in a CrCoNi medium‐entropy alloy (MEA). Theoretically, the authors demonstrated that SRO is thermodynamically favored in HEAs and can be tuned to influence their physical and mechanical properties.^[^ ^15^ ^]^ Zhang et al. ^[^ ^13^ ^]^ reported an increase in strength for CrCoNi MEA after heat treatment at 1200 °C; this increase is attributed to the coupling of SRO hardening and twin‐induced deformation. On the contrary, Inoue et al. ^[^ ^16^ ^]^ showed that the ordering of Co and Ni in a 700 °C heat‐treated CrCoNi MEA does not affect the mechanical properties; the authors argued that the degree of local chemical ordering depends on the heat treatment condition. In fact, they (SRO) are sometimes deleterious—they induce embrittlement and promote abnormal fast crack‐propagation.^[^ ^17^, ^18^ ^]^ As a result, the ability to control their evolution process in HEAs is of interest.

While the existing literature reveals the presence of SRO in different microcrystalline M/HEAs, there are few or no reports on whether or not SRO occurs in nanocrystalline (NC) materials, more so, in NC‐HEAs. Nanocrystalline (NC) materials exhibit remarkable mechanical properties that originate from the large grain boundary (GB) area that inhibits dislocation motions.^[^ ^19^, ^20^, ^21^, ^22^, ^23^ ^]^ However, NC materials are thermodynamically unstable: nanograins rapidly grow even at low homologous temperatures due to the large excess energy of the GBs that drives grain growth, as experimentally and computationally demonstrated in Refs. [24, 25, 26, 27]. The *thermodynamic* stabilization approach proposed by Weissmuller^[^ ^28^ ^]^ and expounded by others^[^ ^29^, ^30^, ^31^, ^32^ ^]^ has become a widely used metallurgical means of stabilizing nanograins; it involves the segregation of dilute solute to the GBs to effectively reduce the excess energy at these interfaces (GBs). It is therefore unclear whether SRO is enhanced or suppressed in GB‐decorated NC‐HEAs.

In our recent work,^[^ ^33^ ^]^ we reported the self‐segregation of Cr and Fe to the GBs of NC‐AlCoCrFe—*self‐stabilization* effect, which stabilized the alloy at high homologous temperatures up to ≈0.52*T_m_* (*T_m_* is the melting temperature) before slight coarsening sets in. The segregated elements have the highest melting point and mixing enthalpy amongst the constituent elements. Furthermore, we showed that the minor addition of a carefully selected solute element (Zr) to NC‐AlCoCrFe—*pseudo‐binary thermodynamic* approach—further enhanced stability up to ≈0.64*T_m_*.^[^ ^23^ ^]^ This is attributed to the coupling effects of Zr segregation (*solute GB segregation effect*) alongside Cr and Fe (*self‐stabilization effect*) to the GBs of the NC‐AlCoCrFe. This study, however, examines the evolution of local chemical structures in such GB‐decorated NC alloy systems due to complex pairwise interactions among the principal constituent elements and the high GB density present. Using high‐energy synchrotron X‐ray diffraction and pair distribution function (HES‐XRD/PDF) for crystal structure and local atomic arrangement analysis, respectively, and atom probe tomography (APT) complemented with Warren‐Cowley (WC) coefficient calculations, we report that GB‐decoration/segregation in NC‐(AlCoCrFe)_100‐x_Zr_x_ suppresses SRO formation.

## 2 Results and Discussion

### 2.1 SRO Prediction Based on Mixing Enthalpy

**Figure 1:**
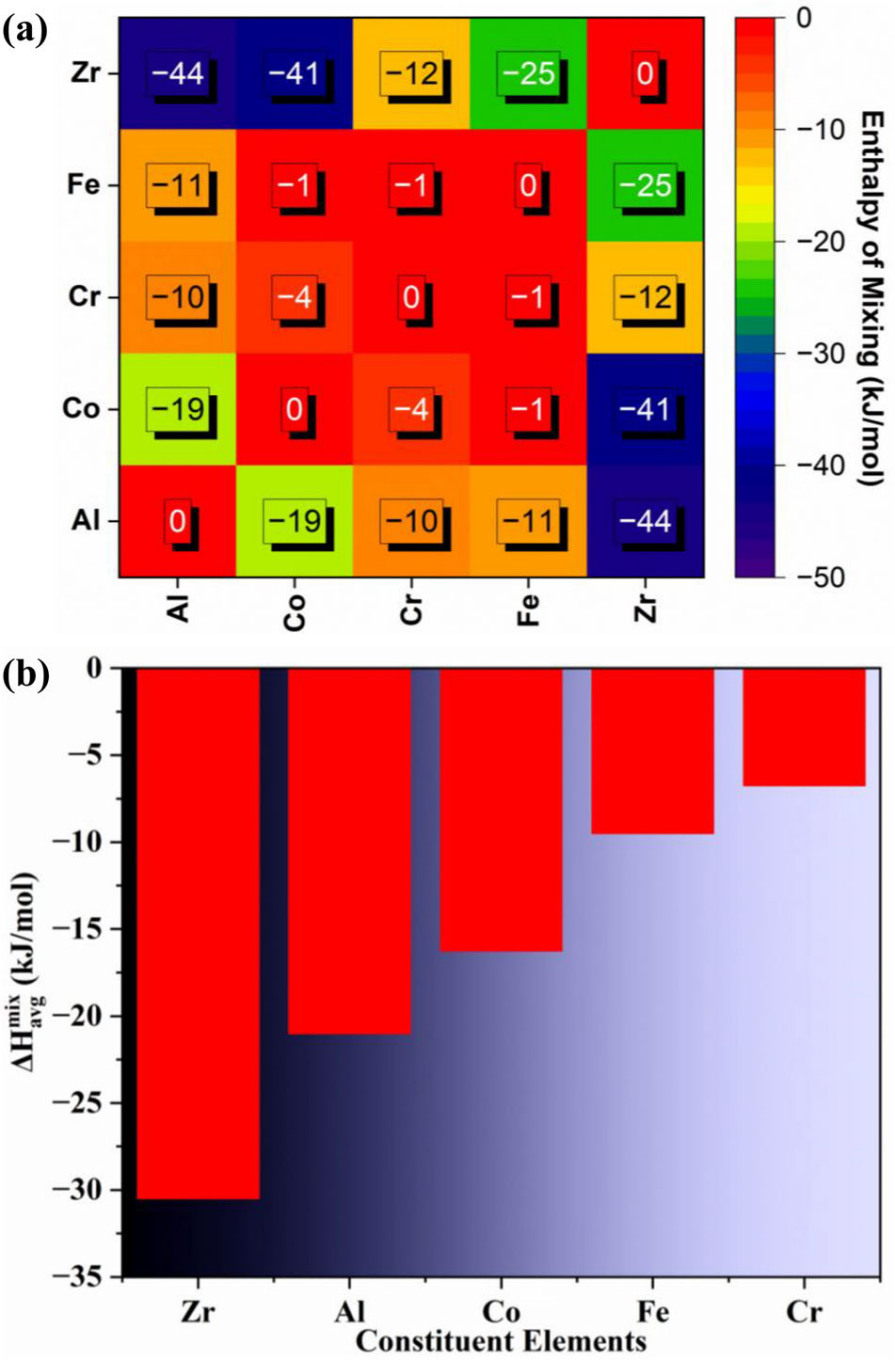
a) Enthalpy of mixing for all the binary pairs in the NC‐HEA, determined from Miedema's model^[^ ^40^ ^]^; b) calculated average enthalpy of mixing of all the elements in the NC‐HEA.

The constituent elements of the alloys under study were carefully selected based on empirical and thermodynamic considerations.^[^ ^23^ ^]^ The NC‐(AlCoCrFe)_100‐x_Zr_x_ were produced from high‐purity Al, Co, Cr, Fe, and Zr elemental powders (≥99.0 %). Following careful selection of the constituent elements, the thermodynamic principles governing the stability of the resulting alloy phases are considered through the Gibbs free energy framework. Besides structural factors like grain size,^[^ ^34^ ^]^ the phase stability of single‐phase alloys is dependent on mixing enthalpy, mixing entropy, and temperature according to Δ*G_mix_* = Δ*H_mix_* − *T*Δ*S_mix_*.^[^ ^35^ ^]^ Compositionally, Δ*H_mix_* governs the formation of SRO^[^ ^36^ ^]^ and atomic segregation phenomena, and as a starting point, we use it (Δ*H_mix_*) to predict the binary elemental pairs that tend to form SRO among the constituent elements. A more positive mixing enthalpy indicates atomic immiscibility, making atomic bond formation among unlike atoms energetically unfavorable; these atoms prefer to segregate to GBs. In contrast, a more negative mixing enthalpy reduces the Gibbs free energy and favors atomic bond formation—SRO formation between unlike atomic pairs.^[^ ^36^, ^37^ ^]^ Based on this principle, the ordering (whether SRO or LRO) formation tendency in (AlCoCrFe)_100‐x_Zr_x_ follows the order Al‐Zr > Co‐Zr > Fe‐Zr > Al‐Co > Cr‐Zr > Al‐Fe > Al‐Cr > Co‐Cr > Co─Fe/Cr─Fe, as demonstrated in **Figure** **1**a. Thus, Al‐Zr has the highest tendency to form SRO, while Co─Fe/Cr─Fe has the highest atomic segregation tendency.

The average enthalpy of mixing, $\Delta H_{avg}^{mix}$—the interaction of each element with all the elements at a time^[^ ^38^ ^]^ is also calculated and shown in Figure 1b to determine which elements have the highest tendency to cluster with other elements. While Al and Zr have relatively low $\Delta H_{avg}^{mix}$, Fe and Cr have higher $\Delta H_{avg}^{mix}$ values; again, Al and Zr have the highest tendency to form SRO, and Cr and Fe possess the highest tendency to undergo segregation. Hence, from Figure 1b (following the criterion that there is a tendency for ordering when Δ*H_mix_* is reduced below a threshold value of −15 kJ mol^−1[^ ^39^ ^]^), and without contemplating the grain size of the alloy in the determination of Δ*H_mix_* and $\Delta H_{avg}^{mix}$, Al, Co, and Zr have the propensity to form SRO.

### 2.2 S/TEM and APT Evaluation of NC‐HEAs in the As‐Milled and Annealed States

#### 2.2.1 Phase Decomposition at 973 K

**Figure 2:**
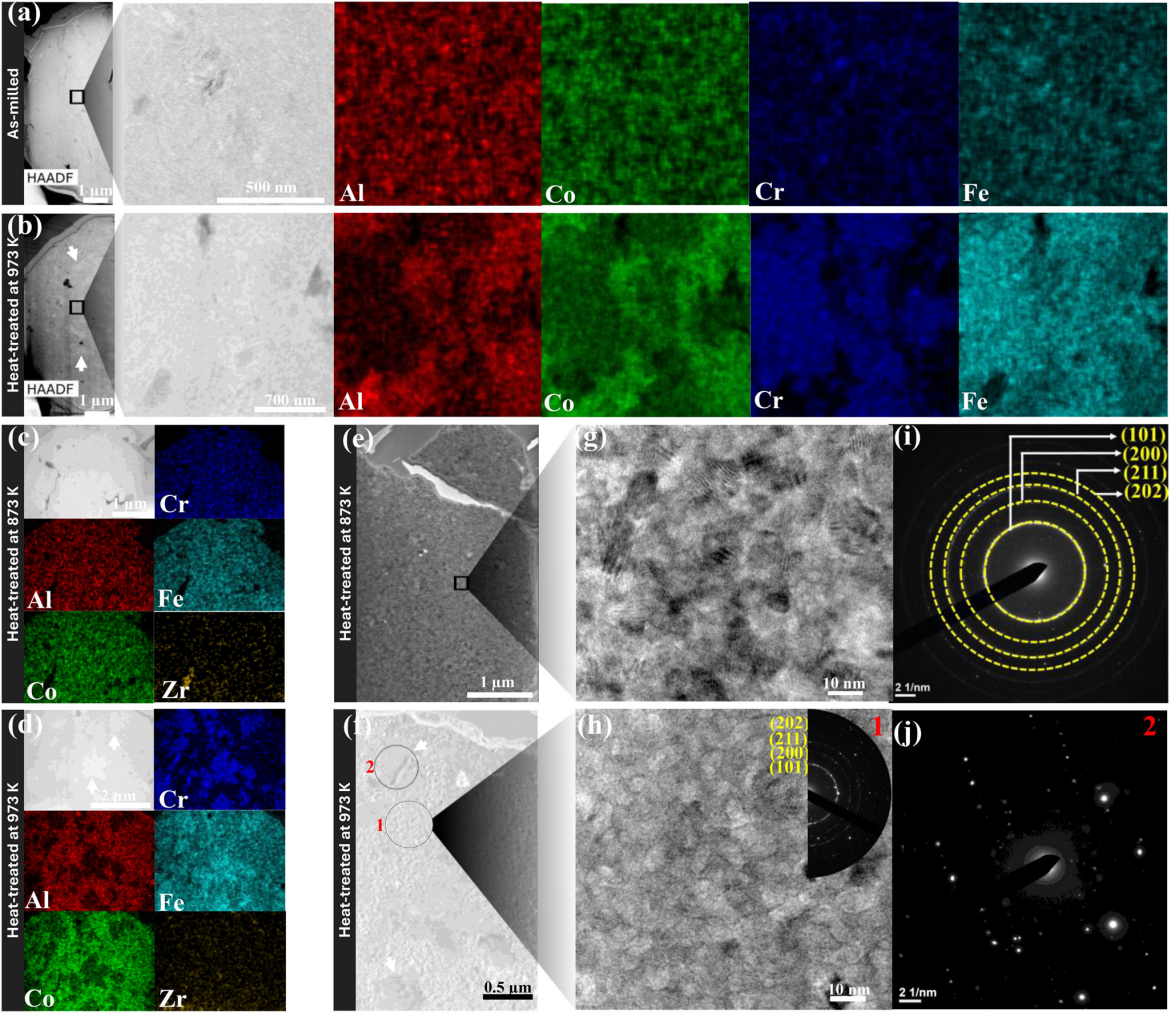
S/TEM micrographs and EDS maps of NC‐(AlCoCrFe)_100‐x_Zr_x_: a,b) are the HAADF images and elemental maps for as‐milled and annealed (at 973 K for 4 h) NC‐AlCoCrFe, respectively; c,d) are the HAADF images and elemental maps for NC‐(AlCoCrFe)_99_Zr_1_ annealed at 873 and 973 K for 4 h, respectively; e–h) are the bright‐field TEM micrographs of NC‐(AlCoCrFe)_99_Zr_1_ annealed at 873 K^[^ ^23^ ^]^ and 973 K for 4 h, respectively; and i,j) are the SAED patterns of (g) and phase‐decomposed region 2 in (f), respectively. White arrows in HAADF and TEM images in (b), (d), and (f) point at the selected phase‐decomposed region, while the inset in (h) is the SAED pattern of the NC region 1 in (f).

The as‐milled NC‐(AlCoCrFe)_100‐x_Zr_x_ are single‐phase solid solutions with BCC crystal structure, while the grains coarsen from ≈7 to ≈16 nm (NC‐AlCoCrFe) and ≈12 nm (NC‐(AlCoCrFe)_98.5_Zr_1.5_), upon annealing at 973 K for 4 h, see Figure S1 (Supporting Information) discussion. The high‐angle‐annular‐dark‐field (HAADF) STEM images and maps for as‐milled NC‐AlCoCrFe in **Figure** **2**a show that all the constituent elements are homogeneously mixed—there is no evidence of segregation or phase decomposition (or LRO). However, phase decomposition of the NC‐AlCoCrFe into Cr─Fe‐rich and Al‐Co‐rich (indicated by white arrows) regions occurs upon annealing at 973 K for 4 h, as shown in Figure 2b. Furthermore, the comparison of bright‐field STEM images and corresponding elemental EDS maps of NC‐(AlCoCrFe)_99_Zr_1_ annealed at 873 and 973 K for 4 h in Figure 2c,d shows similar evidence of Cr─Fe‐rich and Al‐Co‐rich phase decomposition regions at 973 K (indicated by white arrows in Figure 2d).

Phase stability and instability are also evident in the TEM micrographs and SAED patterns shown in Figure 2e,g,i and Figure 2f,h,j, respectively. While NC‐(AlCoCrFe)_99_Zr_1_ annealed at 873 K retains its NC structure (Figure 2e,g, i), the sample annealed at 973 K shows two‐phase regions—a NC structure *region 1* with ≈14 nm in grain size and an obvious darker contrast (higher atomic number elements) GB decoration in Figure 2h, and Cr─Fe‐rich LRO *region 2* with discrete diffraction spots with no rings characteristic of a secondary phase (Figure 2f,j). The Cr─Fe‐rich LRO *region 2* is an ordered intermetallic phase identified in the Thermo‐Calc calculated phase diagram for these alloys in Figure 1c,e of Ref [23]. As the Zr content increases, NC‐(AlCoCrFe)_98.5_Zr_1.5_, the new decomposed phase evolves at a higher temperature (1073 K).^[^ ^23^ ^]^ Given that SROs—initial, incipient ordering tendency that can occur at higher temperatures during annealing or as a material cools down—are ubiquitous in multi‐principal element alloys and are precursors to LROs,^[^ ^41^, ^42^ ^]^ it is envisaged that SRO should form in NC‐(AlCoCrFe)_100‐x_Zr_x_ at least at annealing temperatures just below 973 K, where NC structures are stable (Figure 2a,c,e). In what follows, the as‐milled NC‐AlCoCrFe and NC‐(AlCoCrFe)_98.5_Zr_1.5_, as well as NC‐AlCoCrFe annealed at 773 K and NC‐(AlCoCrFe)_99_Zr_1_ annealed at 873 K, which exhibit nanocrystalline thermal stability, are further examined using APT.

#### 2.2.2 Grain Boundary Segregation at Temperatures Below 973 K

**Figure 3:**
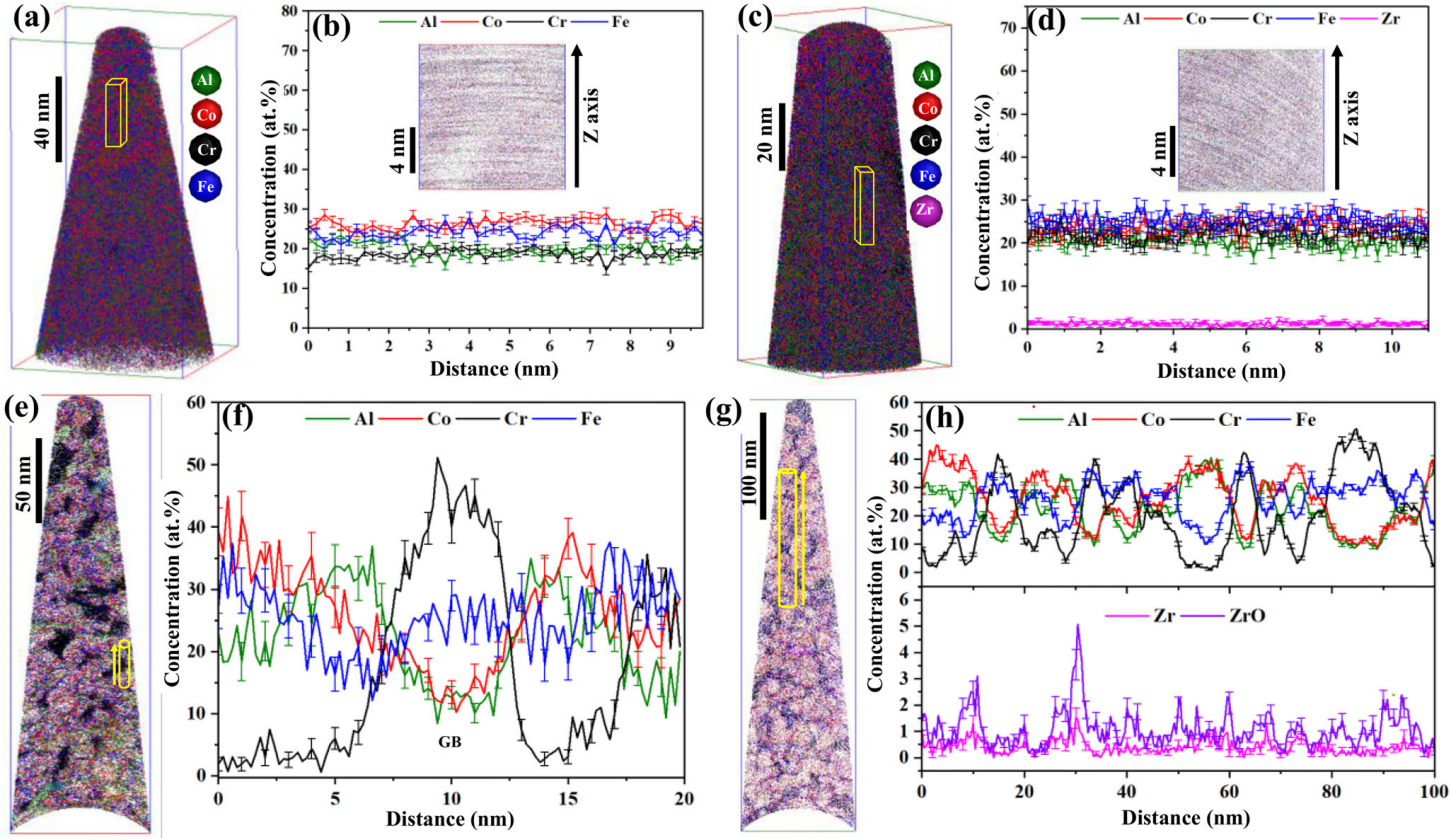
APT reconstruction of NC‐HEAs needles: a) 3D atom map of NC‐AlCoCrFe in the as‐milled state^[^ ^33^ ^]^; b) 1D concentration profile generated along the z‐axis of the inset—5 nm thin slice taken from the marked region in (a)^[^ ^33^ ^]^; c) 3D atom map of NC‐(AlCoCrFe)_98.5_Zr_1.5_ in the as‐milled state^[^ ^23^ ^]^; d) 1D concentration profile generated along the z‐axis of the inset—5 nm thin slice taken from the marked region in (c)^[^ ^23^ ^]^; e) 5 nm thin slice taken from the annealed (773 K for 4 h) NC‐AlCoCrFe needle^[^ ^33^ ^]^; f) 1D concentration profile generated from the cylindrical sub‐volume (marked in (e)) taken across a GB^[^ ^33^ ^]^; g) 5 nm thin slice taken from the annealed (873 K for 4 h) NC‐(AlCoCrFe)_99_Zr_1_ needle^[^ ^23^ ^]^; and h) 1D concentration profile generated from the cylindrical sub‐volume (marked in (g)) across two GBs.^[^ ^23^ ^]^

The nanostructure of NC‐AlCoCrFe and NC‐(AlCoCrFe)_98.5_Zr_1.5_ in the as‐milled state is shown in **Figure** **3**a–d. The APT 3D atom map in Figure 3a shows a homogeneous distribution of all the elements in the NC‐AlCoCrFe—no evidence of segregation in the as‐milled state. A 1D concentration profile in Figure 3b taken along the z‐axis of a 5 nm thin slice (inset in Figure 3b) from a region of interest in Figure 3a shows that the nominal equiatomic composition of all the constituent elements is fairly maintained after mechanical alloying. Similarly, the nominal equiatomic composition of NC‐(AlCoCrFe)_98.5_Zr_1.5_ is maintained in the as‐milled state, as shown in Figure 3c,d. However, the APT reconstruction of NC‐AlCoCrFe annealed at 773 K for 4 h in Figure 3e,f shows GB *self‐segregation* of Cr and Fe; this is due to the high mixing enthalpy of Cr and Fe as established in Figure 1. This self‐segregation effect stabilizes NC‐AlCoCrFe against grain growth even up to a maximum temperature of 873 K.^[^ ^33^ ^]^

Likewise, the APT reconstruction of NC‐(AlCoCrFe)_99_Zr_1_ annealed at 873 K for 4 h in Figure 3g,h shows the *solute GB segregation* of Zr/ZrO alongside the GB *self‐segregation* of Cr and Fe; Zr segregation is due to its negative enthalpy of GB segregation.^[^ ^23^ ^]^ Note that the ZrO ion peak overlaps with Cr and Fe ions in the APT mass spectra in Figure S2 (Supporting Information). Hence, the Cr and Fe ion peaks are contributing to ZrO dominance/counts in the 1D concentration profile in Figure 3h, since Cr and Fe are also segregating with Zr/ZrO at the GBs (see Supporting Information for details of peak overlaps). ZrO also suggests the preferential evaporation of O and Zr species, i.e., the evaporation of Zr as both single ions and molecular ZrO species.^[^ ^43^ ^]^ This is supported by the TEM analysis in Figure 2 and Figure S1 (Supporting Information), which confirms the absence of ZrO phase in the specimens. The two effects—*self‐segregation* of Cr and Fe and *solute GB segregation* of Zr—combine to stabilize the NC‐HEAs further up to 1073 K as established in Ref. [23]. We note that this GB segregation should not be confused with phase separation by spinodal decomposition, which requires no thermodynamic barrier, and more so, there is no miscibility gap in the phase diagram of these alloys (Figure 1c,e of Ref [23]). Although SRO is expected to be a precursor to LRO, as discussed in *Section* *2.2.1*, the observation of GB segregation raises the question of whether SRO is suppressed by the GB segregation events. In the ensuing sections, we examine NC‐(AlCoCrFe)_100‐x_Zr_x_ for both LRO and SRO formation at different annealing temperatures using in situ high‐energy synchrotron XRD/PDF.

### 2.3 In Situ Long‐Range Structural Analyses of the Synthesized NC‐HEAs using HES‐XRD

#### 2.3.1 Crystal Structure and Phase Stability

**Figure 4:**
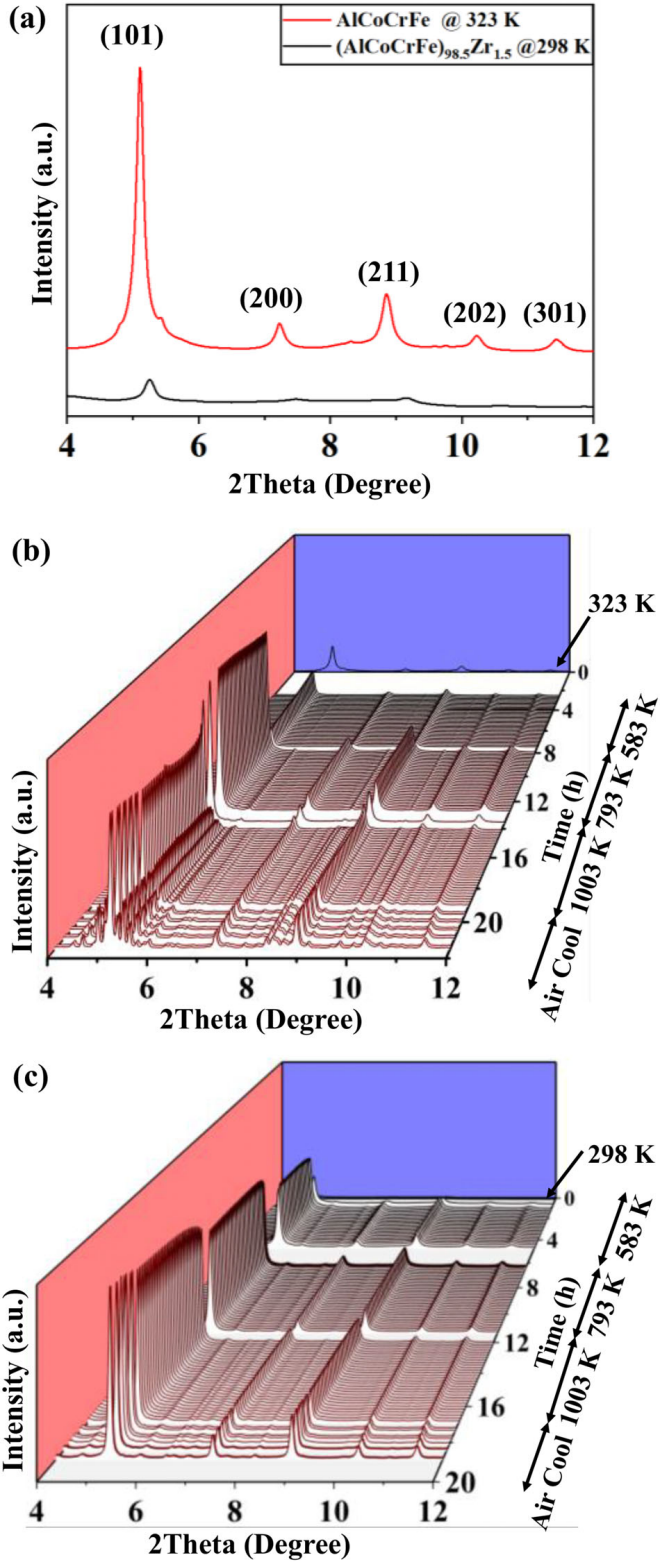
1D diffraction patterns of NC‐AlCoCrFe and NC‐(AlCoCrFe)_98.5_Zr_1.5_: a) NC‐AlCoCrFe and NC‐(AlCoCrFe)_98.5_Zr_1.5_ in the as‐milled state; b) in situ annealed NC‐AlCoCrFe at different temperatures—583 K (≈0.35T_m_), 793 K (≈0.47Tm), and 1003 K (≈0.60T_m_) and air cooled to 423 K (≈0.25T_m_); c) in situ annealed NC‐(AlCoCrFe)_98.5_Zr_1.5_ at different temperatures—583 K (≈0.35T_m_), 793 K (≈0.47Tm), and 1003 K (≈0.60T_m_), and air cooled to 384 K (≈0.23T_m_).

**Figure** **4**a–c shows the in situ HES‐XRD patterns for NC‐AlCoCrFe and NC‐(AlCoCrFe)_98.5_Zr_1.5_ in the as‐milled state and during in situ annealing at 583, 793, and 1003 K; the XRD pattern for the cooling regime is also included. In the as‐milled state, the developed NC‐HEAs are in BCC single‐phase solid‐solution—(101), (200), (211), (202), and (301) peaks, as shown in Figure 4a, and the corresponding 2D HES‐XRD pattern in Figure S3a1 (Supporting Information); this is similar to the observation in Figure S1c,d (Supporting Information), and our prior works.^[^ ^23^, ^33^, ^44^, ^45^ ^]^ A careful inspection of the Bragg peak positions reveals a slight shift to higher 2θ angles with Zr addition. This can be attributed to the non‐uniform compressive lattice strain induced by the large solute atom, Zr, in the NC‐HEA.^[^ ^46^, ^47^ ^]^

A Video S1 (Supporting Information), shows the response of NC‐AlCoCrFe to different annealing temperatures and times. The corresponding 1D diffractograms in Figure 4b, and 2D diffraction pattern in Figures S3a2–a3 and S3b1‐b3 (Supporting Information) show no evolution of new peaks during annealing at 583 and 793 K, a signature of phase stability at these temperatures. One notable feature in Figure 4b is that the peak intensities are unchanged over time at a specific annealing temperature; however, there is an accompanying increase in peak intensity transitioning from 583 K (310 °C) to 793 K (520 °C), which is a characteristic of improved crystallinity,^[^ ^48^, ^49^ ^]^ internal energy release, and grain growth.^[^ ^50^, ^51^ ^]^ Note that in our previous work,^[^ ^33^ ^]^ nanograins are also observed to be stable over time at each annealing temperature up to 873 K, and slight coarsening is only observed at transitioning temperature—773 to 873 K and 873 to 973 K; hence, the increase in peak intensity transitioning from 583 to 793 K in Figure 4b is a result of slight grain growth, in addition to internal energy release and improved crystallinity.

However, increasing the annealing temperature to 1003 K (730 °C) results in the appearance of new peaks and a decrease in peak intensity; new and weak diffraction rings also emerge in the 2D diffraction patterns at the start of annealing at 1003 K as indicated with red arrows in Figure S3c1 (Supporting Information). The new peak intensities become stronger and more visible after 5 h of annealing at 1003 K and subsequent cooling to 423 K (see Figure S3c2, c3, Supporting Information); a signature of phase instability at 1003 K. The strong new peaks/diffraction rings are a close match to Al_5_Co_2_ (Crystallography Open Database (COD) #: 1524269)^[^ ^33^ ^]^; this LRO is in agreement with the prediction in Figure 1. The remaining new peaks/diffraction rings could not be indexed as their intensities are too low.

Meanwhile, the addition of 1.5 at. % Zr to NC‐AlCoCrFe results in substantial phase stabilization after annealing at 583, 793, and 1003 K, and subsequent cooling in air to 384 K, as shown in the Video S2 (Supporting Information), and Figure 4c. Contrary to what is observed in the 2D diffraction pattern of NC‐AlCoCrFe, there are no new diffraction rings associated with the 2D diffraction pattern of NC‐(AlCoCrFe)_98.5_Zr_1.5_ after annealing and cooling, as shown in Figure S3d–f (Supporting Information), except the HCP (100) peak, which is associated with the fairly unmixed Zr, as also established in Figure S1d (Supporting Information). Thus, the addition of 1.5 at. % Zr to the NC‐HEA suppresses new phase formation/evolution at these temperatures.

#### 2.3.2 Grain Stability and Lattice Strain

**Figure 5:**
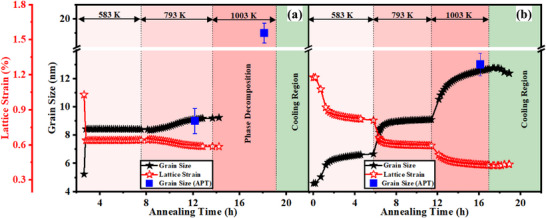
Grain size and lattice strain vs annealing time and temperatures: a) NC‐AlCoCrFe, and b) NC‐(AlCoCrFe)_98.5_Zr_1.5_; grain size determined from APT (when the samples were annealed at 773 and 973 K for 4 h each) are also included (blue square data points) for comparison; these values are also reported in Ref. [33].

The instantaneous grain size and the corresponding lattice strain of NC‐AlCoCrFe (**Figure** **5**a) and NC‐(AlCoCrFe)_98.5_Zr_1.5_ (Figure 5b) are determined from the 1D diffraction patterns in Figure 4b,c. The lattice strain, calculated using the Williamson‐Hall equation,^[^ ^61^ ^]^ generally decreases with increasing grain size. The lattice strain in NC‐(AlCoCrFe)_98.5_Zr_1.5_ (≈1.2 %) is slightly higher than in NC‐AlCoCrFe (≈1 %) in the as‐milled state due to the introduction of the solute atom Zr; doping induces lattice strain.^[^ ^52^ ^]^ Upon annealing, grain size increases, and lattice strain decreases for all NC‐HEAs due to the release of internal strain energy and lattice defects induced by the plastic deformation during milling.^[^ ^53^, ^54^ ^]^ There is a significant decrease in lattice strain between the as‐milled state and the first annealing temperature due to significant internal energy release at the start of annealing. While the lattice strain drop is slow and insignificant between 583 and 793 K annealing temperatures for NC‐AlCoCrFe, it is slightly significant in NC‐(AlCoCrFe)_98.5_Zr_1.5_ between 583 and 793 K annealing temperatures and between 793 and 1003 K annealing temperatures. The lattice strain is fairly stable with annealing time.

Meanwhile, there is an increase in grain size of NC‐AlCoCrFe from ≈5 nm in the as‐milled state to ≈8 nm after annealing at 583 K; the nanograins remained stable at this temperature for 7 h 30 min (≈8 nm) as seen in Figure 5a. Increasing the annealing temperature results in a slight increase in grain size from ≈8 nm at the end of annealing at 583 K to ≈9 nm at 793 K, which also remained stable till the end of annealing after 5 h 36 min. The increase in grain size at the start of each annealing temperature is attributed to the alloy system's attainment of the thermodynamically preferred grain size at these temperatures.^[^ ^23^, ^33^, ^55^ ^]^ However, the onset of phase decomposition when the annealing temperature is raised to 1003 K makes it challenging to determine the instantaneous grain size at this temperature and after cooling to 423 K. Nevertheless, the grain size determined from the APT results for NC‐AlCoCrFe annealed at 773 K (Figure 3e) and 973 K (Ref. [33]) for 4h is ≈9 and ≈19 nm, respectively. These values are represented by blue square data points in Figure 5a, and they confirm that long‐range phase decomposition coincides with nanograin coarsening at 973 K, as reported in Ref. [33].

Meanwhile, there is an initial slight increase in grain size of NC‐(AlCoCrFe)_98.5_Zr_1.5_ from ≈5 nm in the as‐milled state to ≈6 nm after annealing at 583 K as seen in Figure 5b; the nanograins remained stable after annealing for 5 h at 583 K. When the annealing temperature was raised to 793 K, the grain size increased to ≈9 nm and remained stable at that size for 4 h. Unlike NC‐AlCoCrFe, there is nanograin stability after annealing at 1003 K; the grain size slightly increased from ≈9 nm at 793 K to ≈11 nm at 1003 K for 1 h and subsequently ≈13 nm after annealing for 4 h at the same temperature. The final grain size after cooling the sample in air to 384 K is ≈12 nm. Therefore, it can be safely concluded that the solute GB segregation of Zr suppresses LRO (phase decomposition/separation) and grain growth in NC‐(AlCoCrFe)_98.5_Zr_1.5_.

#### 2.3.3 Rietveld Refinement and Lattice Constants

**Figure 6:**
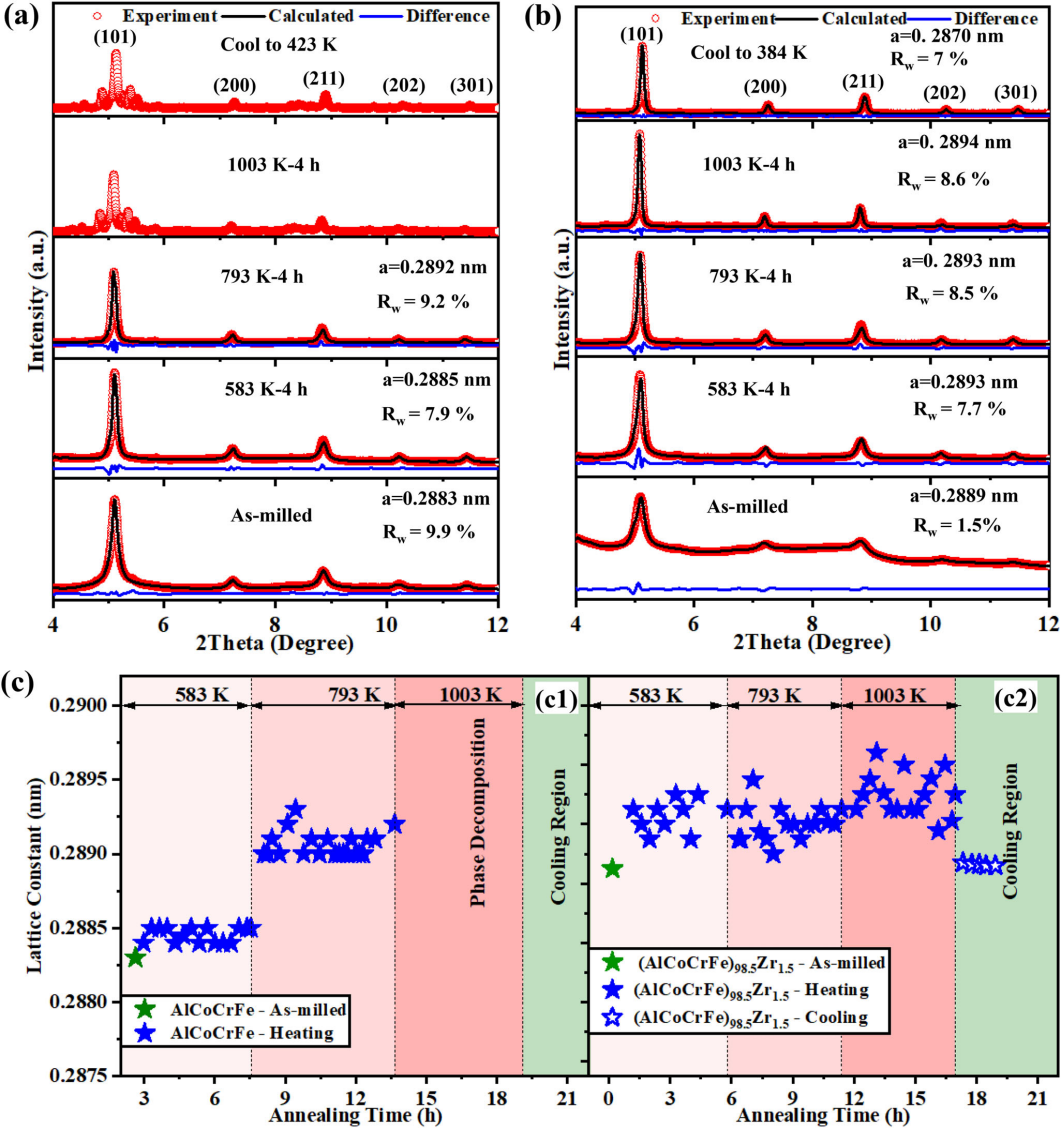
1D X‐ray diffraction pattern and the Rietveld refinement fits as a function of 2θ angle: a) NC‐AlCoCrFe and b) NC‐(AlCoCrFe)_98.5_Zr_1.5_; the associated lattice constant as a function of annealing temperature and time are presented in c)—(c1) NC‐AlCoCrFe and (c2) NC‐(AlCoCrFe)_98.5_Zr_1.5_.

To determine the lattice constants in all the NC‐HEAs and their response to temperature, Rietveld refinement of the HES‐XRD data was first performed using the GSAS II software to fit all the observed data in Figure 4 with a standard BCC structural model (*Im‐3m* space group); the results are given in **Figure** **6**. The observed, calculated, and difference diffraction patterns for NC‐AlCoCrFe and NC‐(AlCoCrFe)_98.5_Zr_1.5_ in the as‐milled and annealed (up to 1003 K for 4 h) states are shown in Figure 6a,b. There is a good agreement between the model and the observed data, with *R_w_* (weighted R‐factor) values <10 %,^[^ ^56^ ^]^ except at high temperature and cooling regimes where phase decomposition sets on in NC‐AlCoCrFe (Figure 6a); the model in these regimes could not be fitted with the observed data and is not included in Figure 6a.

The calculated lattice constants, *a*, after Rietveld refinement for NC‐AlCoCrFe and NC‐(AlCoCrFe)_98.5_Zr_1.5_ are shown in Figure 6c. In the as‐milled state, *the* value for NC‐AlCoCrFe and NC‐(AlCoCrFe)_98.5_Zr_1.5_ are 0.2883 and 0.2889 nm, respectively, as shown in Figure 6c1,c2 (the first data points in green color). It can be observed that the introduction of Zr to the NC‐HEA increases *a*; the increase in lattice constant is attributed to the substitutional replacement of some Al, Co, Cr, and Fe atoms with Zr atoms within the NC‐HEA lattice, as also reported in Ref. [57]. It is worth noting that out of the five alloying elements, Zr has the largest atomic radius, which can increase the overall NC‐HEA lattice constant after alloying; the atomic radii of Al, Co, Cr, Fe, and Zr are 0.143, 0.125, 0.125, 0.124, and 0.160 nm, respectively.^[^ ^23^, ^58^, ^59^ ^]^ During the annealing of NC‐AlCoCrFe at 583 and 793 K (Figure 6c1), *a* increases to 0.2885 and 0.2891 nm, respectively; these values remain fairly constant throughout the annealing period (≈4 h each). The initial increase in lattice constant at the start of each annealing temperature is in agreement with previous works^[^ ^60^, ^61^ ^]^—*a* increases with annealing temperature. The large difference in lattice constant between 583 and 793 K in Figure 6c1 is responsible for the slow drop in lattice strain between these annealing temperatures in Figure 5a. Meanwhile, the lattice constants' stability at these temperatures with time can be attributed to the self‐segregation of Cr and Fe at the GBs of NC‐AlCoCrFe, which self‐stabilized the NC‐HEA system (Figure 3e–h).

For the NC‐(AlCoCrFe)_98.5_Zr_1.5_ annealing at 583 K for 5 h (Figure 6c2), there is an increase in *a* from the as‐milled state (0.2889 nm) to ≈0.2893 nm after 4 h. The lattice constant remains stable and fairly unchanged when the annealing temperature is raised to 1003 K. This is a departure from what is observed in NC‐AlCoCrFe (Figure 6c1), and it is responsible for the drop in lattice strain between the annealing temperatures in Figure 5b. This may be attributed to the GB segregation of Zr in addition to the self‐segregation of Cr and Fe. In other words, the nanograin stabilization by the coupled effect—*self‐segregation* of Cr and Fe and *solute GB segregation* of Zr—also results in the stability of the lattice constant of NC‐HEAs. Because SRO typically leads to lattice contraction rather than expansion during annealing,^[^ ^62^ ^]^ the increase in lattice constants (at the start of annealing temperatures) and their stability during annealing support a lack of SRO in NC‐HEAs. Next, the ex situ HES‐PDF data for NC‐(AlCoCrFe)_98.5_Zr_1.5_ at 973 K, where no phase decomposition occurs, are analyzed to probe the local arrangements of atoms in the as‐milled state and after annealing at different temperatures.

### 2.4 Short‐Range Structural Analysis by HESPDF

**Figure 7:**
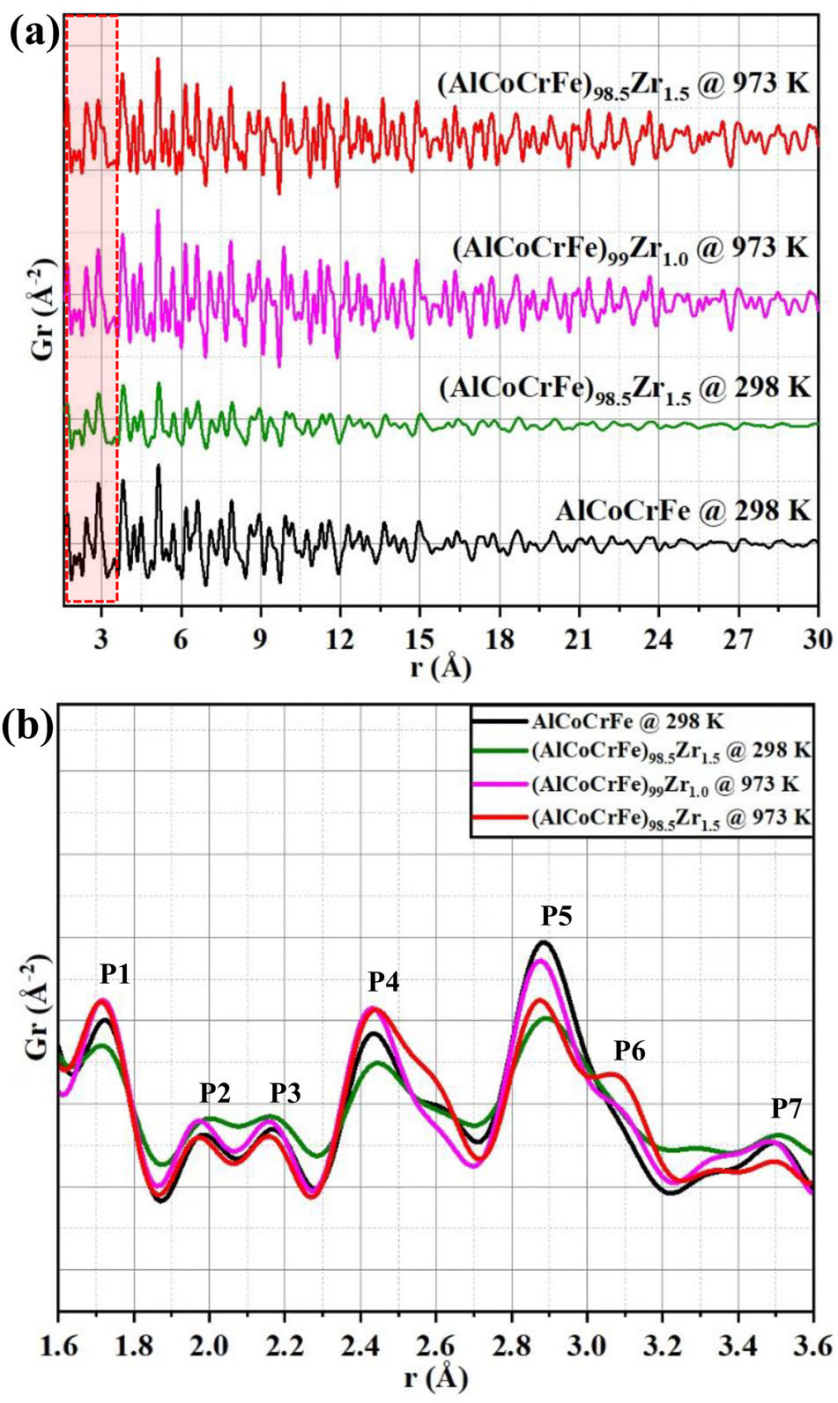
Ex situ X‐ray PDF profiles of the NC‐HEAs: a) PDF profiles at higher r values, up to 30 Å of NC‐AlCoCrFe and NC‐(AlCoCrFe)_98.5_Zr_1.5_ in the as‐milled state, and NC‐(AlCoCrFe)_99_Zr_1_ and NC‐(AlCoCrFe)_98.5_Zr_1.5_ after annealing at 973 K for 4 h; and b) PDF profiles at lower r values (1.6 to 3.6 Å) for NC‐AlCoCrFe and NC‐(AlCoCrFe)_98.5_Zr_1.5_ HEAs in the as‐milled state, and NC‐(AlCoCrFe)_99_Zr_1_ and NC‐(AlCoCrFe)_98.5_Zr_1.5_ after annealing at 973 K for 4 h.

Short‐range structural insights into the as‐milled NC‐AlCoCrFe and NC‐(AlCoCrFe)_98.5_Zr_1.5_ and the associated changes with annealing temperature were obtained through analysis of the ex situ X‐ray PDF in real space, at higher *r* range (up to 30 Å), as shown in **Figure** **7**; NC‐(AlCoCrFe)_99_Zr_1_, which also exhibits improved phase and grain stability, is included. The PDF method retains both the Bragg and diffuse scattering from the sample, and it contains information on the local atomic disorder and local atomic correlations; it offers the probability of finding a pair of atoms at (*r*) distance separation.^[^ ^54^, ^63^, ^64^, ^65^ ^]^ The peak positions in PDF are characteristic of the interatomic distances, while the peak areas and widths give information on the coordination number (or coordination shell) and atomic thermal vibrations, respectively.^[^ ^54^, ^66^ ^]^ The X‐ray PDF profiles with higher *r* range (up to 30 Å) for NC‐AlCoCrFe and NC‐(AlCoCrFe)_98.5_Zr_1.5_ in the as‐milled state are shown in Figure 7a; the PDF profiles for NC‐(AlCoCrFe)_99_Zr_1_ and NC‐(AlCoCrFe)_98.5_Zr_1.5_ after annealing at 973 K for 4 h are also included.

The minor addition of Zr up to 1.5 at. % leads to slight damping of the peak intensity, especially at higher r values (>15 Å) in the as‐milled state; this is characteristic of atomic correlation damping—loss of LRO and crystallinity with Zr addition.^[^ ^68^, ^69^ ^]^ There is no observable or significant peak shift with Zr addition in the as‐milled state. However, there is a discernible difference between the PDF peaks of NC‐(AlCoCrFe)98.5Zr1.5 in the as‐milled state and after annealing at 973 K for 4 h; the PDF peak intensities increase after annealing.

The PDFs at low *r* values (1.6–3.6 Å) containing the first seven coordination shells are further probed to discern differences in peak intensity and to correlate the different bond pairs present in the NC‐HEAs with peak positions in the as‐milled state and after annealing, as shown in Figure 7b. The peaks within the selected *r* range are numbered P1 to P7 for ease of description, and the bond lengths of all the binary pairs are presented in Table 1. The first peak (P1) is observed at 1.72 Å, and it corresponds to the Al─O bond. P2 occurs at 1.98 Å, corresponding to either Co─O, Cr─O, Fe─O, or Fe─Fe bonds; since the amount of the interstitial element oxygen is insignificant in the synthesized NC‐HEA, P2 is most likely a Fe─Fe bond. While P3 is observed at 2.16 Å and corresponds to the Zr─O bond, P4 occurs at 2.44 Å and is close to Al─Co, Al─Cr, Co─Cr, and Co─Fe bonds, as shown in Table 1. The major peak (P5) is observed at 2.89 Å, which closely matches Co─Zr, Al─Al, and Cr─Cr bonds. In the as‐milled state, there is no peak at 3.07 Å (indicated with P6 for later discussion). The 7^th^ coordination shell (P7) is observed at 3.5 Å, and it is close to the Fe─Zr bond.

**Table 1:** PDF peak positions and their corresponding nearest neighbors and bond lengths.^[^ ^67^ ^]^

Peaks	*r* Value (Å)	Nearest Neighbors	Bond Lengths (Å)
P1	1.72	Al – O	1.92
P2	1.98	Co – O Cr – O Fe – O Fe – Fe	1.96 2.01 – 2.06 2.06 2.05 – 2.08
P3	2.16	Zr – O	2.12
P4	2.44	Al – Co Al – Cr Co – Cr Co – Fe	2.47 2.46 – 2.76 2.47 2.49
P5	2.89	Co – Zr Al – Al Cr – Cr	2.79 – 3.00 2.88 – 2.94 2.74 – 2.81
P6	3.07	Cr – Zr Zr – Zr	2.95 – 3.06 3.10
P7	3.5	Fe – Zr	3.47

In the as‐milled state, NC‐(AlCoCrFe)_98.5_Zr_1.5_ exhibits a slight peak broadening than NC‐AlCoCrFe across all coordination shells. Note that the PDF peak width may be a function of two components, *static* and *dynamic* atomic displacement within the crystal.^[^ ^70^ ^]^ Static atomic displacement is related to the dislocation density, local lattice strains, and surface defects present in the sample, while dynamic atomic displacement is entirely related to the thermal vibrations of atoms about their ideal site.^[^ ^70^ ^]^ We, therefore, attribute the peak broadening observed in the as‐milled state to static atomic displacement. In a substitutional solid solution, all atoms are displaced from their idealized lattice sites, regardless of their distance from the substitution point; the interatomic bonds store strain energy from the lattice distortion.^[^ ^71^ ^]^ In the present work, the introduction of large‐sized Zr into the NC‐AlCoCrFe induced a local strain field due to the Zr atom substitution of Al, Co, Cr, and Fe atoms. This effect would broaden the PDF peaks.^[^ ^70^ ^]^ At 973 K, we studied the effect of Zr amount on the local atomic arrangement of AlCoCrFe: the increase in Zr content from 1.0 at. % (NC‐(AlCoCrFe)_99_Zr_1_) to 1.5 at. % (NC‐(AlCoCrFe)_98.5_Zr_1.5_) results in a corresponding decrease in peak intensity in coordination shells P5 and P7. This observation indicates a general decrease in the nearest‐neighbor bond lengths with increasing Zr content. Thus, the addition of Zr into the NC‐HEA, which segregates at the GBs after annealing at 973 K for 4 h, helps stabilize the bonds, especially the nearest neighbor bonds corresponding to P5 and P7.

Examining the effect of temperature and, by extension, GB segregation, there is a slight peak shift to lower *r* values and slight peak broadening across all coordination shells of NC‐(AlCoCrFe)_98.5_Zr_1.5,_ especially the dominant shells after annealing at 973 K for 4 h. The peak broadening observed can be attributed to the dynamic atomic displacement of atoms about their lattice positions—the thermal motion of atoms, while the peak shift to lower *r* values is attributed to the decrease in the respective bond lengths.^[^ ^72^ ^]^ The decreased bond length between the as‐milled state and 973 K agrees with the decrease in lattice constants from the as‐milled state to higher annealing temperature, 1003 K, observed in Figure 6c2. The broadening of P5 in the as‐milled state is associated with disordering of the NC‐HEA; however, the 6th coordination shell (P6), which corresponds to Cr─Zr and Zr─Zr bonds, is now more distinct in NC‐(AlCoCrFe)_98.5_Zr_1.5_ after annealing at 973 K. At this temperature, there is a higher concentration of Cr, Fe, and Zr at the GBs than in the matrix due to segregation, as shown in Figure 3g, h. The evolution of such a peak (P6) in a PDF profile is typically attributed to SRO,^[^ ^36^, ^72^ ^]^ leading to the question of whether this distinct peak evolution should indeed be attributed to short‐range ordering of Cr‐Zr or Zr‐Zr atoms, or the GB segregation of Cr, Fe, and Zr (Figure 3e, g). In what follows, we investigate the presence of SRO in the synthesized NC‐HEAs, both in the as‐milled state and after annealing, using APT and WC coefficient calculations.

### 2.5 Investigating the Presence of SRO in NC‐HEAs from APT Data and Warren–Cowley Coefficient Analysis

Experimental techniques that provide adequate crystallographic information at the atomic level, such as X‐ray scattering and TEM, have been utilized to characterize SRO in alloys;^[^ ^13^, ^73^, ^74^, ^75^ ^]^ however, they cannot image 3D information of complex structures such as SRO. Recently, TEM has been used to characterize SRO in MEA and HEA;^[^ ^74^, ^76^ ^]^ however, this technique provides only 2D projections of SRO. As an analytical tool with high sensitivity (10–100 ppm) and near‐atomic spatial resolution, APT, in principle, can detect 3D elemental distribution—providing an opportunity to detect SRO. However, this technique has some drawbacks, too: its spatial resolution is low, and its detection efficiency is limited;^[^ ^86^, ^87^ ^]^ these limitations prevent it from precisely revealing atomic ordering processes in the < 1 nm regime. Furthermore, complications arise as the alloy complexity and number of defects, such as GBs, increase.^[^ ^77^ ^]^ Recent studies have adopted machine learning/simulation‐assisted APT approaches that utilize SDMs to reveal crystallographic information and the statistical distributions of different elements in binary and medium‐entropy alloys within APT data to detect SROs successfully.^[^ ^16^, ^78^, ^79^, ^80^, ^81^ ^]^ In the current work, we adopt statistical methods—SDM from APT and WC coefficient quantification approaches to a core complex multicomponent HEA.

#### 2.5.1 SRO Evaluation by APT Analysis

With SDM analysis, possible recurring patterns (SRO) in a randomly distributed solid solution can be revealed along the chosen crystallographic direction.^[^ ^16^ ^]^ In pure metals, clear, discernible poles are typically observed in the ion hit map, facilitating the determination and calibration of the image compression factor during reconstruction. However, because the NC‐HEA under study is made up of five different elements, Al, Co, Cr, Fe, and Zr, all with slightly different evaporation fields,^[^ ^80^ ^]^ there is some disorder during evaporation. This makes it difficult to identify clear crystallographic features from the detector hit maps, where reliable SDM histogram data can be generated. To overcome this challenge, a thin slice of the voltage curve—the applied voltage required to induce field evaporation—is made (region of interest), and the corresponding ions are displayed in the detector hit map; faint crystallographic poles become visible on the detector hit map with this approach. To achieve an appropriate reconstruction, the *x* and *y* detector coordinates of the crystallographic poles, the shank angle, and the initial radius of the needle are used to calculate and calibrate the image compression factor. These parameters are iteratively varied to obtain the appropriate interplanar spacing; this is similar to the approach adopted in Ref. [82].

**Figure 8:**
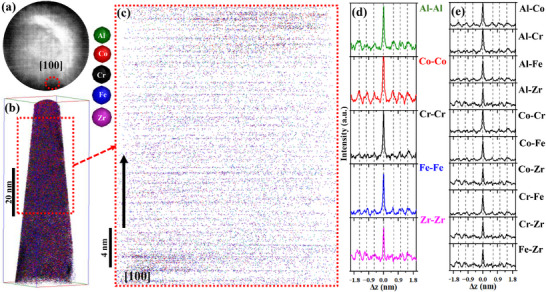
APT data of NC‐(AlCoCrFe)_98.5_Zr_1.5_ in the as‐milled state: a) detector hit map of 160 million ions impact after slicing the voltage curve which contains a low‐density [100] crystallographic pole; b) 3D atom map of ions of the constituent elements; c) 2D projection of the reconstruction after calibration; it shows the atomic planes; d) z‐SDMs of same element pairs; and e) z‐SDMs of cross elements pairs.

**Figure** **8**a shows the 2D detector hit map of ≈160 million ion impacts after slicing the voltage curve of the APT data of solid‐solution NC‐(AlCoCrFe)_98.5_Zr_1.5_ in the as‐milled state. A low‐density crystallographic pole corresponding to [100] is observed. Regions that experienced significant trajectory aberrations are the low‐density regions (dark region in Figure 8a), and these are areas where atomic planes can be imaged in the reconstructed APT dataset.^[^ ^83^ ^]^ Unlike pure metals and binary alloys that have well‐defined crystallographic poles in the detector hit map, only a few faint crystallographic poles can be discerned in Figure 8a due to the significant disorder during evaporation of the different alloying elements in the NC‐HEA. The 3D atom map of NC‐(AlCoCrFe)_98.5_Zr_1.5_ is shown in Figure 8b, which shows that all constituent elements are uniformly distributed.

Figure 8c is a 2D projection of the calibrated reconstruction in Figure 8b; the direction indicated by the arrow corresponds to the [100] direction, and it contains BCC‐resolved planes. The reconstruction was calibrated to have an interplanar spacing of ≈0.290 nm; this is close to the TEM results in Figure S1f (Supporting Information). The z‐SDM for Al─Al, Co─Co, Cr─Cr, Fe─Fe, and Zr‐Zr pairs along [100] direction is presented in Figure 8d. One notable feature is that the z‐SDMs for the same pair elements are identical—species peak‐to‐peak distances are equal for all elemental pairs, an indication that all the constituent elements have a strong tendency to occupy each other's lattice sites.^[^ ^84^ ^]^ The z‐SDMs for the cross‐species elemental pairs are also shown in Figure 8e; the peak‐to‐peak distance is identical and equal to the same pair of elements, and it shows the average distribution of one element atom relative to the other element atom along the [100] direction.^[^ ^85^ ^]^ These observations are an indication that a homogeneous solid solution is achieved^[^ ^80^ ^]^—the random distribution of all the constituent elements in the as‐milled state. Additionally, the peak intensities do not vary across the atomic planes over a large distance, <1 nm, except for the central peak (which is normalized); confirmation that there is no deviation from a random solid solution. The deviation from a random distribution of the constituent elements is evidence of the presence of chemical ordering in the NC‐HEA.^[^ ^16^, ^86^ ^]^ By classification, SRO is established when the deviation in histogram peak intensity spans below 1 nm; similarly, histogram peak intensity deviation for medium‐range and long‐range order spans 1 to 5 nm and more than 5 nm, respectively.^[^ ^3^ ^]^ Because there are no deviations at any distance, Δ*z*, in Figure 8d,e, we can safely conclude that there is no evidence of SRO in as‐milled NC‐(AlCoCrFe)_98.5_Zr_1.5_.

**Figure 9:**
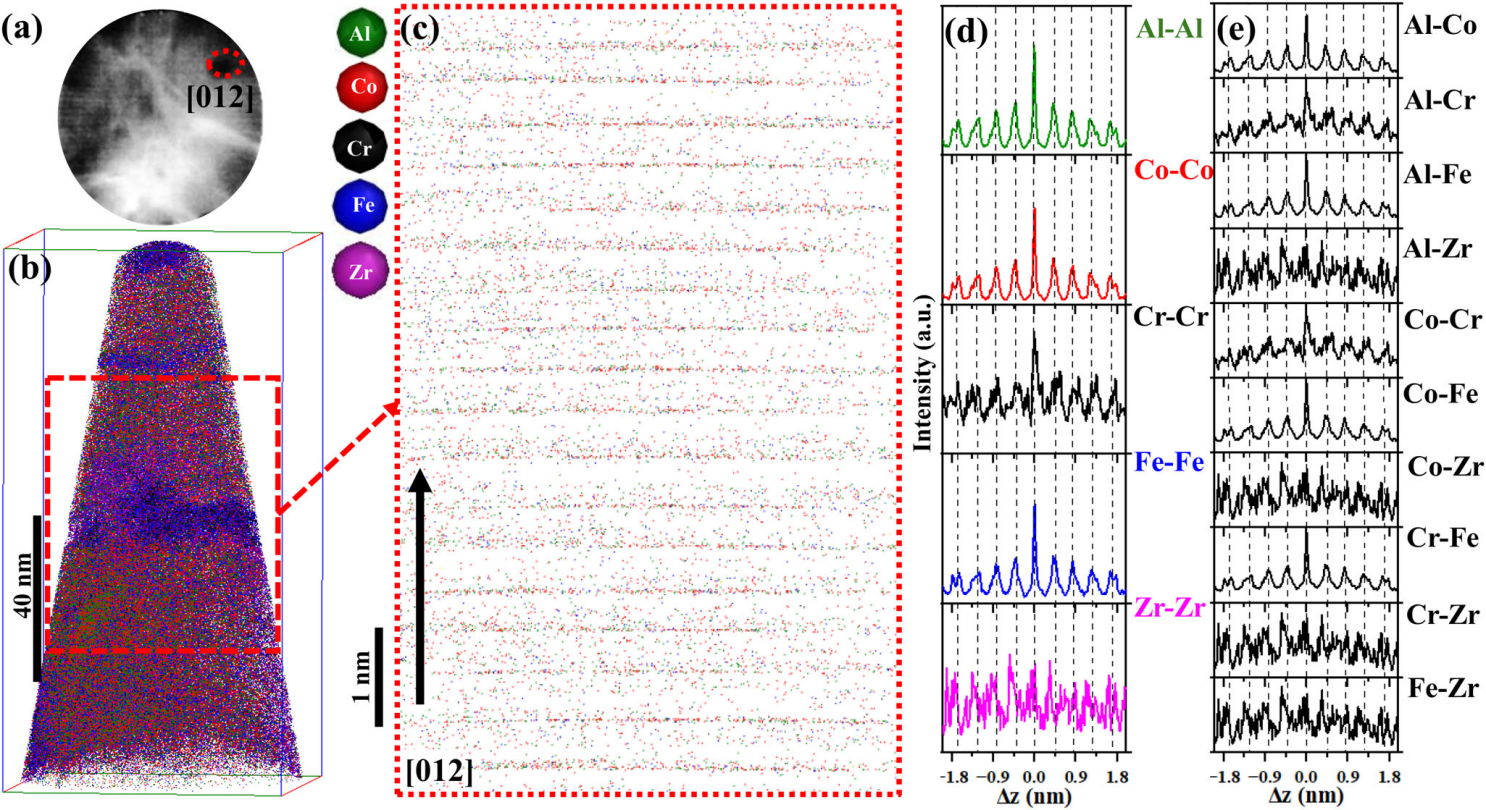
APT data of NC‐(AlCoCrFe)_98.5_Zr_1.5_ sample after annealing at 973 K for 4 h: a) detector hit map of 90 million ions impact after slicing the voltage curve which contains a low‐density [012] crystallographic pole; b) 3D atom map of ions of the constituent elements that shows GBs are decorated; c) 2D projection of the reconstruction after calibration; it shows the atomic planes; d) z‐SDMs of same element pairs; and e) z‐SDMs of cross elements pairs.

Following the similar analysis used for as‐milled NC‐(AlCoCrFe)_98.5_Zr_1.5_ in Figure 8, we assessed possible SRO at elevated temperatures, 873 (0.53*T_m_*) and 973 K (0.59*T_m_*) for NC‐(AlCoCrFe)_99_Zr_1_ and NC‐(AlCoCrFe)_98.5_Zr_1.5,_ respectively. **Figure** **9**a shows the 2D detector hit map of 90 million ion impacts after slicing the voltage curve of the APT data of the annealed NC‐(AlCoCrFe)_98.5_Zr_1.5_. The crystallographic pole (low‐density region) marked with a red circle corresponding to [012] direction is observed and indexed. The 3D atom map, presented in Figure 9b, reveals that GBs are enriched with Cr, Fe, and Zr after annealing, as established in Ref. [23] and Figure 3f,h. The atomic planes of Al, Co, Cr, Fe, and Zr ions perpendicular to the [012] direction within the annealed NC‐(AlCoCrFe)_98.5_Zr_1.5_ are clearly shown in the 2D projection of the calibrated reconstruction (see Figure 9c); atomic planes can clearly be visualized. The interplanar spacing is ≈0.320 nm, close to what is reported in the TEM results in Figure S1l (Supporting Information).

**Figure 10:**
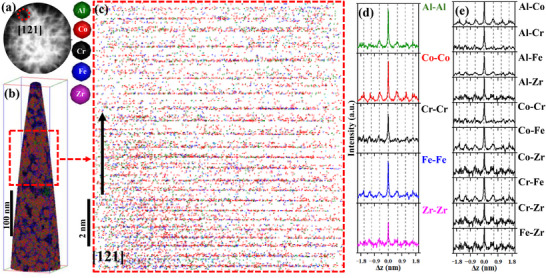
APT data of NC‐(AlCoCrFe)_99_Zr_1_ after annealing at 873 K for 4 h: a) Detector hit map of 20 million ions impact after slicing the voltage curve which contains a low‐density [121] crystallographic pole; b) 3D atom map of ions of the constituent elements that shows GBs are decorated; c) thin slice of elemental ions after calibrating the reconstruction; it shows the atomic planes; d) z‐SDMs of same element pairs; and e) z‐SDMs of cross elements pairs.

Figure 9d details the z‐SDMs for the same element pairs—Al─Al, Co─Co, Cr─Cr, Fe─Fe, and Zr‐Zr pairs. Like what is observed in the as‐milled state (Figure 8d), the interplanar spacing for all the same elemental pairs is equal, indicating no deviation from the random distribution even after annealing—the elements are still uniformly distributed even in the matrix. Figure 9e shows the z‐SDMs for the cross‐element pairs that are identical to the same element pairs. There is a non‐oscillating peak height across the atomic planes, except between the central peak and the 1st peak. This indicates that, despite Cr, Fe, and Zr segregating at the GBs, the remaining elements in the matrix are uniformly mixed with Al and Co, which become significantly present after annealing. That is, segregation of Cr, Fe, and Zr at the GBs after annealing suppressed ordering in the matrix; note that segregation can alter the SRO parameters in the bulk.^[^ ^87^ ^]^ To confirm this unique observation, the APT data for NC‐(AlCoCrFe)_99_Zr_1_ after annealing at 873 K for 4 h are also analyzed and are showcased in **Figure** **10**.

The spatial distribution of the 20 million elemental ions on the detector after slicing the voltage curve is shown in Figure 10a; a [121] crystallographic pole that contains resolved planes is observed. The 3D atom map is also shown in Figure 10b. As observed in Figures 8c and 9c, the 2D projection of the calibrated reconstruction clearly shows atom planes perpendicular to the [121] direction. The species peak‐to‐peak separation distance of the z‐SDMs histograms for the same element and cross‐element pairs are identical (interplanar spacing of ≈0.300 nm)—atoms of the constituent elements tend to occupy each other lattice sites after annealing at 873 K. This confirms that despite the segregation of Cr, Fe, and Zr at the GBs after annealing which changed the chemical potential in the matrix, self‐organization of same element or cross element species within the matrix is still not favored. The APT results show that GB segregation of Cr, Fe, and Zr in the nanocrystalline HEA state suppresses potential and predicted elemental pair orderings (Figure 1) in the matrix after annealing. By extension, the evolution of P6 in Figure 7b is safely concluded as a signature of GB segregation rather than SRO, as occasionally interpreted.^[^ ^72^ ^]^ As a next step, we perform a WC coefficient analysis of the calibrated reconstructed APT (experimental) data for Zr‐doped NC‐HEA in the as‐milled and heat‐treated state to further quantitatively confirm the absence or presence of SRO.

#### 2.5.2 The Warren Cowley Coefficients Analysis

**Figure 11:**
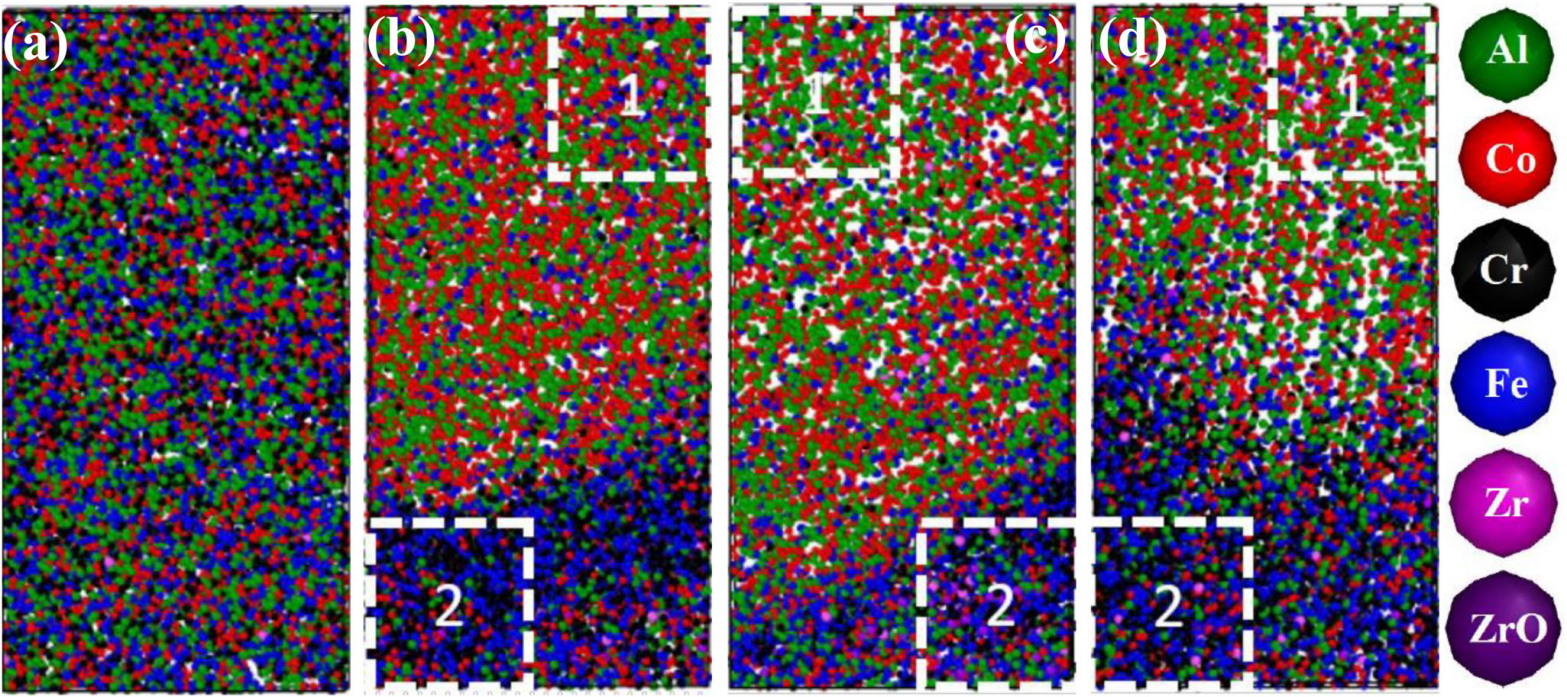
Calibrated APT reconstruction: a) 5 × 5 × 10 nm^3^ region of interest (ROI) of NC‐(AlCoCrFe)_98.5_Zr_1.5_ in as‐milled state, (b‐d) A 5 × 5 × 10 nm^3^ ROIs b) 1, c) 2, and d) 3, taken from the matrix through to the GBs of the annealed (at 873 K) NC‐(AlCoCrFe)_99_Zr_1_. The Warren‐Cowley coefficients analysis is performed on the entire ROIs, within the matrix (1), and the GB (2).

**Figure 12:**
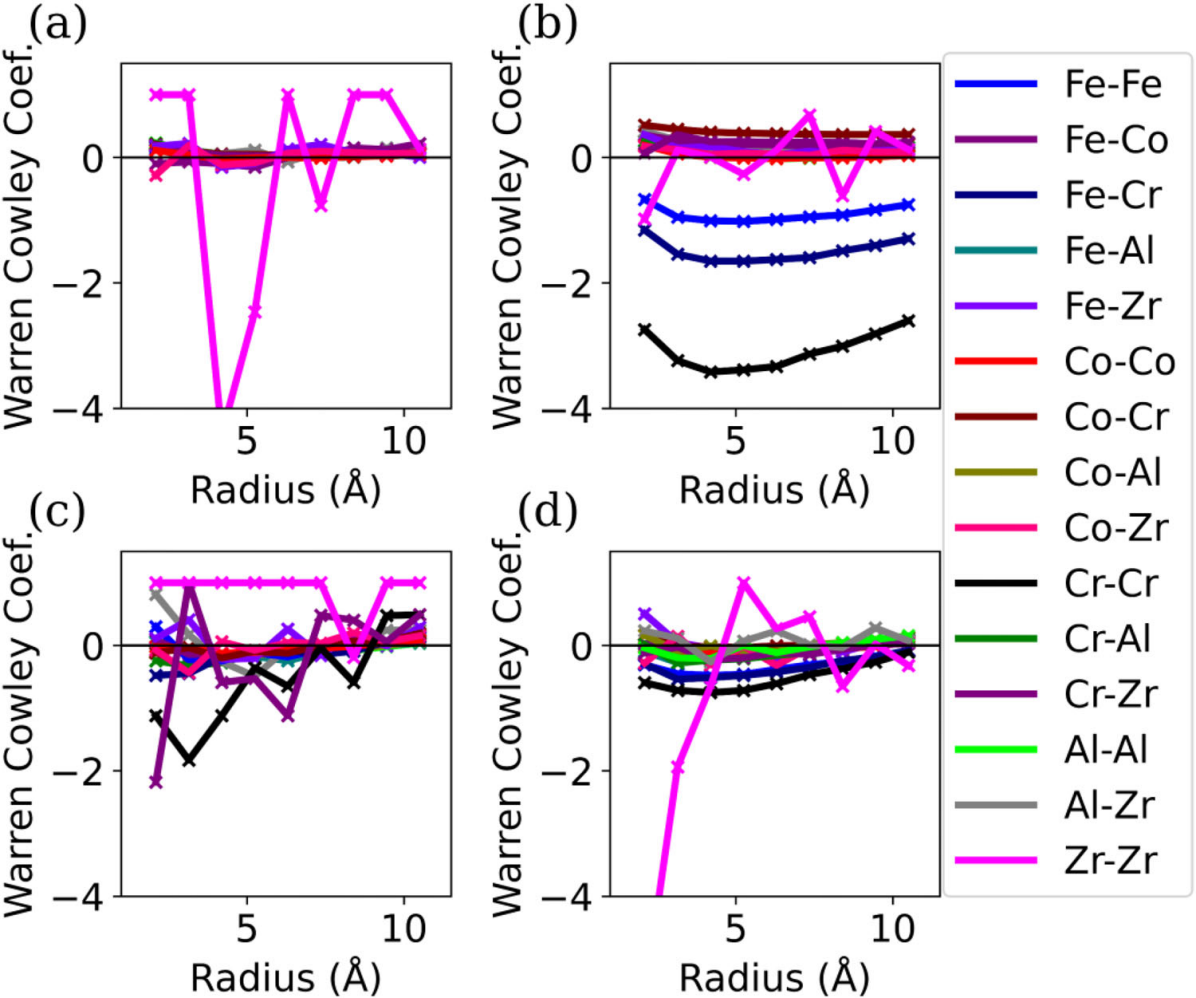
Spatially resolved WC coefficients: a) ROI for as‐milled NC‐(AlCoCrFe)_98.5_Zr_1.5_ in Figure 11a, and annealed NC‐(AlCoCrFe)_99_Zr_1_, b) entire ROI 1 of Figure 11b; c) ROI 1 of Figure 11b taken solely from the matrix; d) ROI 1 of Figure 11b taken solely from the GB. For interpretation of the references to color in this legend, the reader is referred to the web version of this article.

**Figure 13:**
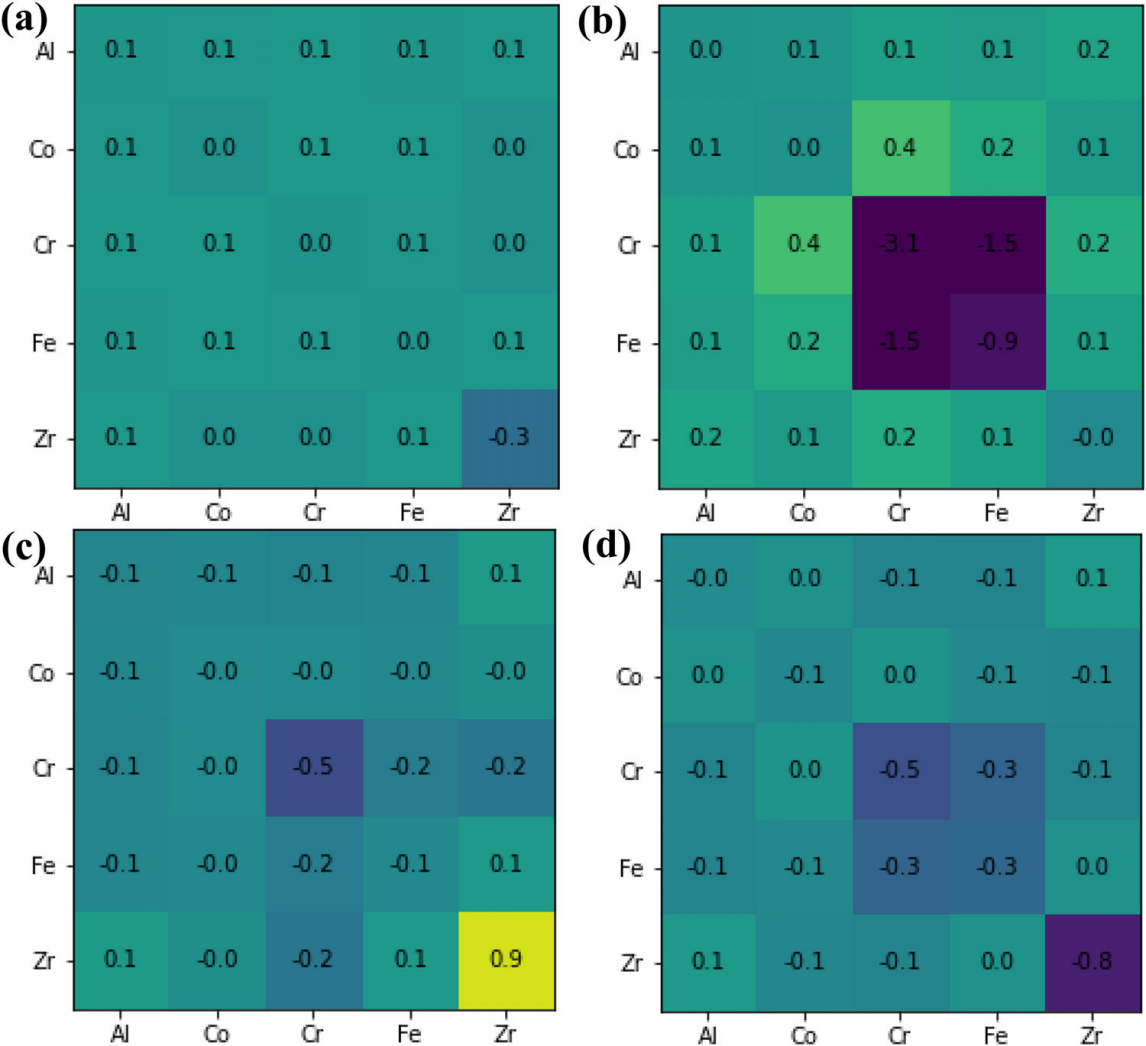
Average WC coefficients for as‐milled NC‐(AlCoCrFe)_98.5_Zr_1.5_ and annealed NC‐(AlCoCrFe)_99_Zr_1_: a) Entire ROI as‐milled NC‐(AlCoCrFe)_98.5_Zr_1.5_ in Figure 11a; b) entire ROI 1 of annealed NC‐(AlCoCrFe)_99_Zr_1_ in Figure 11b; c) ROI 1 of Figure 11b taken solely from the matrix of annealed NC‐(AlCoCrFe)_99_Zr_1_; d) ROI 1 of Figure 11b taken solely from the GB of annealed NC‐(AlCoCrFe)_99_Zr_1_.

The calibrated APT reconstruction for site‐specific WC coefficient calculations in **Figure** **11** shows the defined ROI from the as‐milled Zr‐doped alloy (Figure 11a), and ROIs 1 to 3 taken from the matrix through the GB (Figure 11b–d) of annealed NC‐(AlCoCrFe)_99_Zr_1_. The associated spatially resolved WC coefficients and the averaged WC coefficients are also shown in **Figure** **12** and **13**, respectively. It is important to point out that the low concentration of Zr atoms in the measurements (< 20 atoms of Zr) introduces sampling artifacts that lead to a large noise in the Zr subplots and anomalies, especially noticeable for the subregions in Figure 12a–d. This introduces uncertainty in making conclusions about Zr ordering within the samples. The spatially resolved WC coefficients in Figure 12a, along with the low magnitude of the average WC coefficients observed in the as‐milled NC‐(AlCoCrFe)_98.5_Zr_1.5_ (Figure 13a), demonstrate a lack of SRO, which is consistent with the APT result. However, the mean WC coefficients for Cr─Cr (‐3.1), Cr─Fe (‐1.5), and Fe─Fe (‐0.9) from the entire ROI 1 (Figure 11b) of annealed NC‐(AlCoCrFe)_99_Zr_1_ are more negative than other elemental pairs, as shown in Figure 13b. This points to the strong interactions between Cr and Fe. Even though we observe Fe segregation alongside Cr and Zr (Figure 3g,h), the Fe─Fe and Cr─Fe interactions are smaller compared to the Cr─Cr, as shown in Figure 12b and 13b; this is due to the anti‐clustering tendency of the Fe atoms, which has been reported in NC Fe‐Cr alloys.^[^ ^88^ ^]^

To deconvolve the effect of GB segregation in the annealed NC‐(AlCoCrFe)_99_Zr_1_, the spatial and averaged WC coefficients were computed for the matrix (Figures 12c and 13c) and at the GB (Figures 12d and 13d), respectively. While the WC coefficients for these subregions show some preferential Cr─Cr interactions (WC = ‐0.5)—preferential clustering of Cr atoms in the vicinity of other Cr atoms, the average magnitude of the WC coefficients is less than one‐sixth that of the overall ROI 1. This indicates that there is no ordering in the subregions. The Cr─Cr WC coefficients for the matrix and GB regions are both negative, with the minima at radii ≈3 and 4 Å, respectively, as shown in Figure 12c,d, suggesting preferential clustering. However, because Cr, Fe, and Zr preferentially segregate to the GBs, their concentration within the matrix is lower, as shown in the 1D concentration profile in Figure 3h. Cr has the highest GB segregation tendency among the three species, as established in Ref. [23], and therefore has a low bulk concentration. This introduced uncertainty in calculating the WC coefficients for the Cr─Cr pair, especially in the matrix. Note that even though Cr, Fe, and Zr segregate at the GBs, these species are uniformly distributed in the GB area as the magnitude of the averaged WC coefficients for Cr─Cr, Fe─Fe, and Cr─Fe in Figure 13d are within the range for random distribution as reported for some of the elemental binary pairs in Ref. [89]. The other binary pairs—Al─Al, Al─Co, Al─Cr, Al─Fe, Al─Zr, Co─Co, Co─Cr, Co─Fe, Co─Zr, Cr─Zr, Fe─Zr show a lack of SRO, with WC coefficients near zero—signifying their uniform/random distribution in the matrix and GBs. The average WC coefficient for the Cr‐Zr pair in Figure 13 is also near zero, indicating the random mixing of these elements; as a result, the new peak (P6) in the PDF profile of Figure 7b cannot be related to Cr─Zr ordering but rather to the GB segregation of Cr, Fe, and Zr. Since the intensity of peak P6 increases with increasing Zr content, its evolution could be primarily driven by Zr segregation at the GBs. A similar analysis is performed for ROIs 2 and 3 (Figures S4 and S5, Supporting Information), and it also shows that there is no SRO in the NC‐HEA annealed at 873 K. The WC coefficient analysis quantitatively illustrates that the SRO tendency between Cr and Fe atoms is weaker within the GB and matrix areas than in the entire ROIs because Cr, Fe, and Zr segregate to the GBs.

### 2.6 GB‐segregation is Energetically Favored Over SRO in Nanocrystalline‐HEA Structure

The results from HES‐XRD/PDF, APT, and the Warren‐Cowley coefficients analysis altogether did not reveal any detectable SRO in either the as‐milled or annealed NC‐HEAs studied. These findings contradict the SRO atomic pair‐forming tendency prediction from the mixing enthalpy principle discussed in Section 2.1. This discrepancy shifts our attention to the possible influence of the alloys’ grain structure on SRO, and not just the composition that the mixing enthalpy/Gibbs‐free energy criterion contemplates. Specifically, the absence of SRO in the current study can be attributed to both the composition of the alloy systems that constitute GB‐segregating elements and the nanocrystalline structure of the alloy, where the high GB density and short diffusion path to GBs—inter‐GB distance^[^ ^33^ ^]^ favor GB segregation of Cr, Fe, and Zr. The excess energy and diffusion at the GBs are higher than in the matrix,^[^ ^90^ ^]^ and as a result, GB segregation of these elements (Cr, Fe, and Zr) is more favored than their clustering within the space‐restricted nanograins. In other words, in a coarse‐grained HEA system (long diffusion path to GBs), SRO is likely to be favored at the expense of GB‐segregation; this explains the perceived contradictions with prior experimental or computational works that are reported in Refs. [13, 36, 89, 91, 92, 93]. For instance, the ordering of the Cr‐Co pair near GB is reported in the CrCoNi MEA despite Ni GB segregation,^[^ ^91^ ^]^ which may be due to compositional difference (compared to the current study), and also the focus on single GB geometry in the computational framework, which is not representative of a bulk nanocrystalline structure. From a thermodynamic standpoint, the Gibbs free energy for SRO formation is on the order of ‐10 meV per atom (≈‐1 kJmol^−1^),^[^ ^87^ ^]^ which is much higher than the GB segregation enthalpies for Cr (≈‐23 kJmol^−1^), Fe (≈‐8 kJmol^−1^), and Zr (≈‐8 kJmol^−1^) in the AlCoCrFe‐Zr system.^[^ ^23^ ^]^ As a result, the activation energy for GB segregation of Cr, Fe, and Zr in NC‐HEA should be lower than for in‐grain SRO formation. We posit that once GB segregation sets on, the chemical potential within the matrix is altered, thereby disfavoring the formation of SROs.^[^ ^87^ ^]^

## 3 Summary and Conclusion

Short‐range ordering generally occurs in binary and multi‐principal element alloys, which sometimes can be deleterious to the alloy system. Hence, it is imperative to have control over their evolution process. Using AlCoCrFe‐Zr as a model alloy, SRO or LRO formation tendency follows the enthalpy of mixing prediction order Al─Zr > Co─Zr > Fe─Zr > Al─Co > Cr─Zr > Al─Fe > Al─Cr > Co─Cr > Co─Fe/Cr─Fe, as shown in Figure 1. While LRO formation aligns with the expectation of this enthalpic prediction, we show that SRO is suppressed in as‐milled and GB‐decorated NC‐(AlCoCrFe)_100‐x_Zr_x_ using HRTEM, HES‐XRD/PDF, APT, and Warren‐Cowley coefficient analysis. The following specific conclusions can be drawn:

The introduction of the large‐sized solute element, Zr, to the NC‐AlCoCrFe increases the lattice constant and also induces non‐uniform comprehensive lattice strain in NC‐HEA, especially in the as‐milled state, due to the substitutional replacement of some Al, Co, Cr, and Fe atoms with Zr atoms within the NC‐HEA lattice.

There is an increase in lattice constants when the temperature is increased from the as‐milled state to 583 K and from 583 to 793 K in NC‐AlCoCrFe, but the lattice constants remain stable with increasing annealing times at each of these temperatures. Meanwhile, the lattice constant is stable and fairly unchanged during in situ annealing of NC‐(AlCoCrFe)_98.5_Zr_1.5_ across all annealing times and temperatures—583, 793, and 1003 K. This observation indicates the absence of SRO.

After annealing the NC‐HEA at 973 K for 4 h, the nearest neighbor bond length decreases with increasing Zr amount up to 1.5 at. %, as Cr, Fe, and Zr segregate to the GBs of the NC‐HEA.

Short‐range ordering is absent in the synthesized NC‐HEA; it is also suppressed in NC‐(AlCoCrFe)_99_Zr_1_ and NC‐(AlCoCrFe)_98.5_Zr_1.5_ after annealing at 873 and 973 K, respectively, for 4 h. This is due to the early onset of energetically favored Cr, Fe, and Zr segregation to the NC‐HEA GBs.

The expected correlation between the emergence of a new peak in the PDF profile during annealing and the formation of SRO deviates in this study. Instead, the evolution of the new PDF peak results from Cr, Fe, and Zr GB segregation.

Warren‐Cowley coefficient analysis shows that the preferential location of Cr, Fe, and Zr atoms within several nearest neighbor distances of each other is overshadowed by their segregation to the GB, and the other alloying components show no evidence of SRO. Although these elements are significantly present in the GB area, there is no SRO at the GB interface based on the WC coefficient analysis.

The findings of this study highlight the role of GB segregation in governing the evolution of SRO in nanocrystalline multi‐principal element alloys. A high GB density increases the energy of the system while also reducing the boundary‐to‐boundary diffusion path that promotes the segregation of “segregable” elements—Cr, Fe, and Zr to the GBs. This segregation, in turn, suppresses short‐range ordering within the matrix. When elements with a tendency to cluster in the matrix preferentially segregate at the GBs, the local matrix chemistry is altered, thereby disfavoring the formation of SRO.

## 4 Experimental Section

### Mechanical Alloying

Milling of the constituent element powders was carried out in a planetary ball mill (PBM‐04, MicroNano Tools, Canada) in an Argon atmosphere. While Zirconia vacuum grinding jars, stainless‐steel balls, and stainless‐steel jackets were used as milling media, 1 weight percent (wt.%) ethanol was used as the process control agent, and a ball‐to‐powder ratio of 10:1 was maintained throughout the milling process. First, Al, Co, Cr, and Fe powders in equiatomic ratio were milled for 20 h at 350 rpm to develop a BCC single‐phase solid solution of NC‐AlCoCrFe (i.e., Zr = 0 at. %); this alloy serves as the “solvent” for the developed *pseudo‐binary thermodynamic* approach established in Ref. [23]. The minor concentration of Zr (solute element) varied up to 1.5 at. % was added to the “solvent”, followed by an additional 20 h of milling to develop AlCoCrFe, (AlCoCrFe)_99_Zr_1_, and (AlCoCrFe)_98.5_Zr_1.5_ single‐phase BCC solid solution in a nanocrystalline state. Alloy sampling and characterization were carried out intermittently throughout the alloying process. Details on the starting powders and the milling parameters/process are found in Refs. [44].

### Heat Treatment and Characterization of the NC‐HEAs Samples

For ex situ microstructural analyses, the as‐milled NC‐HEA powders were annealed based on the observations from the in situ XRD measurements in Refs. [23, 33], in a tube furnace (LINDBERG SOLA BASIC+) for 4 h at each annealing temperature. The annealing temperatures were 773 and 973 K for NC‐AlCoCrFe, 873 and 973 K for NC‐(AlCoCrFe)_99_Zr_1_, and 973 K for NC‐(AlCoCrFe)_98.5_Zr_1.5_. The morphology of the particles and the elemental distribution of the as‐milled and annealed powder were determined by scanning electron microscopy (SEM) using a Bruker NanombH (Berlin, Germany) operating at 20 keV, with an XFlash 5030 energy‐dispersive spectroscopy (EDS) detector. The S/TEM analysis of the as‐milled and annealed samples was performed using a ThermoFisher Scientific X‐FEG Talos 200X, operating at an acceleration voltage of 200 keV coupled with a super‐X Energy‐Dispersive X‐ray Spectroscopy (EDS) detection system. Following a standard in situ lift‐out procedure, the ThermoFisher Scientific Helios G5 Dual‐Beam focused ion beam (FIB) was employed to prepare the TEM lamella. The Ga+ beam voltage was systematically decreased to 2 keV while the lamella thickness was gradually reduced to ≈50 nm. Post‐processing of the HRTEM images was performed using CrysTBox (open‐source) and DigitalMicrograph (GATAN)^[^ ^94^ ^]^ software packages to index diffraction spots and to generate 2D fast Fourier transform (FFT) and inverse FFT (IFFT).

The phase and local atomic ordering in the as‐milled and annealed samples were determined using HES‐XRD and PDF operating in transmission mode at the 28ID‐2 (XPD) beamline of National Synchrotron Light Source II (NSLS‐II), Brookhaven National Laboratory. In situ heating, using a hot air blower, was performed on powder samples packed into 0.1 mm thick quartz capillaries at various temperatures and times for both NC‐AlCoCrFe and NC‐(AlCoCrFe)_98.5_Zr_1.5_ samples. NC‐AlCoCrFe was heat treated at 583 (≈0.35*T_m_*) for 7 h 30 min, 793 (≈0.47*T_m_*) for 5 h 36 min, and 1003 K (≈0.60*T_m_*) for 5 h, and air‐cooled to 423 K (≈0.25*T_m_*). NC‐(AlCoCrFe)_98.5_Zr_1.5_ was heat treated at 583 (≈0.35*T_m_*) for 5 h, 793 (≈0.47*T_m_*) for 4 h, and 1003 K (≈0.60*T_m_*) for 4 h, and air‐cooled to 384 K (≈0.23*T_m_*), where *T_m_* is the melting temperature. A flat‐panel X‐ray detector, the Dexela 2923 CMOS with a 75 x 75 µm^2^ pixel size, was used to capture scattered X‐rays. The incident X‐ray energy was 68.2 keV with a beam dimension (size) of ≈0.25 mm wide and ≈0.25 mm high. The sample‐to‐detector distance and the detector's tilts with respect to the X‐ray beam were calibrated using powdered lanthanum hexaborate (LaB_6_–NIST SMR660c). A 0.1‐second detector acquisition time was set. Averaging multiple frames was done to improve the count statistics. Using the PyFAI software, the resulting 2‐D data were transformed into a 1‐D pattern.^[^ ^95^ ^]^ The grain size (*D*) and lattice strain (ε) were calculated using the Scherrer and Williamson‐Hall equations, respectively.^[^ ^44^, ^96^ ^]^ For post‐processing of the HES‐XRD data, Rietveld refinement was performed using the General Structure Analysis System (GSAS) II software.^[^ ^97^ ^]^ The PDF data were generated by Fourier transformation of the structural function, S(Q), using the PDFgetX3 software.^[^ ^98^ ^]^ SRO in materials generally forms between 0.35*T_m_* and 0.55*T_m_*, and has been observed in multicomponent CoCrNi and CrMnFeCoNi alloys annealed at a temperature range of 0.35–0.51*T_m_* and 0.37–0.54*T_m_*, respectively.^[^ ^62^, ^99^ ^]^ As such, it was posited that the selected annealing temperature range in this work was within the “SRO‐induced temperature window” in the investigated NC‐HEA systems.

APT was used to study the 3D distribution of the constituent elements in the as‐milled and annealed samples at the atomic scale.^[^ ^100^ ^]^ Preparation of needle APT samples was carried out following a standard in situ lift‐out procedure, and details of the steps have already been reported in Refs. [23, 33]. The fabricated specimens were analyzed using the CAMECA Local Electrode Atom Probe (LEAP) 5000 XS. Field evaporation of the specimens was enabled by a high electric potential at the tips in ultra‐high vacuum and cryogenic conditions (10^−11^ torr and ≈30 K). The specimen's apex was pulsed by a 355 nm‐wavelength UV laser (20 pJ, 250 kHz) to control the evaporation of ions. The voltage was then gradually increased to maintain an average ion detection rate of 0.005 ions per pulse (0.5%). Reconstruction of the APT data and generation of spatial distribution maps (SDMs) were done using the AP Suite 6.1 software from CAMECA. The open‐source software ImageJ^[^ ^101^ ^]^ was used to measure average grain sizes from TEM micrographs and APT‐reconstructed needles.

### Warren‐Cowley Coefficient Quantification

Warren‐Cowley (WC) coefficients were used to quantify SRO in multicomponent systems.^[^ ^87^ ^]^ The WC coefficient between species *i* and *j* at a distance *m* is $\alpha_{ij}^{m}=1-\frac{p_{ij}^{m}}{c_{i}c_{j}}$, where $p_{ij}^{m}$ is the probability that an *i* and *j* atom are a distance *m* apart, and *c_i_* and *c_j_* are the concentrations of species *i* and *j*. As a rule of thumb, a WC coefficient less than zero means two species are found at a given distance more often than predicted for random ordering, and a coefficient greater than zero indicates that the two atoms will repel each other. In this work, site‐specific WC coefficients were calculated using the trajectories from calibrated APT reconstruction for the defined region of interest (ROI). Both average WC coefficients and spatially resolved values were computed starting at the nearest neighbor distance of 2 Å with bin sizes of 1 Å up to a cut‐off of 10 Å.

## Conflict of Interest

The authors declare no conflict of interest.

## Supporting information



Supporting Information

Supplemental Video 1

Supplemental Video 2

## Data Availability

The data that support the findings of this study are available from the corresponding author upon reasonable request.
