# Supplementary material for: Grain Boundary Segregation Suppresses Local Short‐Range Ordering in Nanocrystalline High‐Entropy Alloys
Source: Adv Sci (Weinh). 2025 Nov 30;13(6):e15510. doi: 10.1002/advs.202515510 (PMC12866862; doi:10.1002/advs.202515510)
Supplement: Supplementary file 1 — Supporting Information [file ADVS-13-e15510-s002.docx]

**Grain Boundary Segregation Suppresses Local Short-Range Ordering in Nanocrystalline High-Entropy Alloys**

Moses A. Adaan-Nyiak^1,*^, Mack Cleveland^2^, Benjamin Hewitt^1^, Ericmoore Jossou^3,4^, Mehmet Topsakal^5^, Simerjeet K. Gill^5^, Sooyeon Hwang^6^, Kim Kisslinger^6^, Ahmed A. Tiamiyu^1,*^

*^1^Department of Mechanical and Manufacturing Engineering, University of Calgary, 2500 University Drive NW, Calgary, Alberta, T2N 1N4, Canada.*

*^2^Department of Materials Science and Engineering, Massachusetts Institute of Technology, Cambridge, MA, 02139, USA*

*^3^Department of Nuclear Science and Engineering, Massachusetts Institute of Technology, Cambridge, MA, 02139, USA*

*^4^Department of Electrical Engineering and Computer Science, Massachusetts Institute of Technology, Cambridge, MA, 02139, USA*

*^5^Nuclear Science and Technology Department, Brookhaven National Laboratory, Upton, NY, 11973, USA.*

*^6^Center for Functional Nanomaterials, Brookhaven National Laboratory, Upton, NY 11973, USA*

**Corresponding author*: [moses.adaannyiak@ucalgary.ca](mailto:moses.adaannyiak@ucalgary.ca); [ahmed.tiamiyu@ucalgary.ca](mailto:ahmed.tiamiyu@ucalgary.ca)

**SUPPLEMENTARY INFORMATION**

**Elemental Distribution, Crystal Structure, Grain Size, and *d*-spacing Evaluation**

Sample SEM-EDS maps for NC-AlCoCrFe in the as-milled state and after annealing at 773 K for 4 h are shown in Figures S1a and b: all the constituent elements are uniformly distributed with no agglomeration of the particle after heat treatment. The TEM nanostructure evaluation of AlCoCrFe and NC-(AlCoCrFe)_98.5_Zr_1.5_ in the as-milled state and after annealing at 973 K for 4 h is presented in Figures S1c-l. The high-resolution TEM (HRTEM) micrographs in Figures S1c and d show that both HEAs in the as-milled state are nanocrystalline, each with an average size of ~7 nm. The synthesized NC-HEAs are single-phase solid-solutions with a BCC crystal structure as determined by the analyzed selected area electron diffraction (SAED) patterns in Figures S1c and d insets. The SAED pattern of NC-(AlCoCrFe)_98.5_Zr_1.5_ in Figure S1d exhibits a (100)-HCP ring, which can be attributed to the fairly unmixed Zr. The IFFT lattice images for NC-AlCoCrFe and NC-(AlCoCrFe)_98.5_Zr_1.5_ processed from the FFT of the yellow-marked regions in Figures S1c and d are displayed in Figures S1e and f, respectively: they qualitatively and quantitatively (*d-spacing*) show that the extent of lattice distortion is more severe in NC-(AlCoCrFe)_98.5_Zr_1.5_ (0.272 nm) than in NC-AlCoCrFe (0.233 nm) due to the presence of large-sized solute atom, Zr, in the former [1]. This observation aligns with the proposed core effect of HEAs, the “severe lattice distortion effect” [2].

**Figure S1**: **Micro-and Nanostructure characterization of NC-AlCoCrFe and NC-(AlCoCrFe)_98.5_Zr_1.5_ in the as-milled state and after annealing at 773 K and 973 K for 4 h.** SEM-EDS images for NC-AlCoCrFe **(a)** as-milled state, and **(b)** annealed at 773 K; HRTEM micrographs for (**c**) NC-AlCoCrFe and (**d**) NC-(AlCoCrFe)_98.5_Zr_1.5_ in the as-milled state, and the inverse fast Fourier transform of selected grain—rectangular marked areas in (c) and (d)—are presented in **(e)** NC-AlCoCrFe and **(f)** NC-(AlCoCrFe)_98.5_Zr_1.5_; **(g)** HRTEM of NC-AlCoCrFe annealed at 973 K; **(h)** fast Fourier transform of two selected areas (marked 1 and 2 in (g)), each across two adjacent grains; and **(i)** the inverse fast Fourier transform of selected grain—rectangular marked area in (g); and **(j)** HRTEM of NC-(AlCoCrFe)_98.5_Zr_1.5_ annealed at 973 K, **(k)** fast Fourier transform of two selected areas (marked 1 and 2 in (j)), each across two adjacent grains, and **(l)** the inverse fast Fourier transform of selected grain—rectangular marked area in (j).

Annealing of NC-AlCoCrFe and NC-(AlCoCrFe)_98.5_Zr_1.5_ at 973 K for 4 h results in slight nanograin coarsening: average grain sizes for annealed NC-AlCoCrFe and NC-(AlCoCrFe)_98.5_Zr_1.5_ are ~16 and ~12 nm, respectively, as reported in our prior works [3][4]. The FFT diffractogram of two selected areas, each across two adjoining grains in NC-AlCoCrFe (numbered 1 and 2 in Figure S1g) and NC-(AlCoCrFe)_98.5_Zr_1.5_ (numbered 1 and 2 in Figure S1j), is displayed in Figures S1h and k, respectively: while the boundaries in both alloys exhibit some level of crystallinity, there are fewer diffraction spots for NC-AlCoCrFe than in NC-(AlCoCrFe)_98.5_Zr_1.5_. As solute segregation induces crystallinity at GBs [5], the higher diffraction spots in NC-(AlCoCrFe)_98.5_Zr_1.5_ may be attributed to the combined segregation of Zr and self-segregation of Cr and Fe at the GB as seen in Figure 3. For NC-AlCoCrFe, the IFFT of a selected nanograin (marked with a yellow rectangle in Figure S1g) obtained from the FFT in Figure S1i shows an increase in *d-spacing* (0.297 nm) compared to the as-milled state (0.233 nm in Figure S1e) due to the temperature effect, i.e., *d-spacing* increases with increasing temperature [6]. Similarly, the *d-spacing* for NC-(AlCoCrFe)_98.5_Zr_1.5_ increases to 0.291 nm after annealing, as seen in Figure S1l. Conclusively, GB-segregation during annealing in both NC-HEAs is accompanied by an increase in *d-spacing*.

**APT Mass Spectra Analysis**

**Figure S2**: Mass spectra of the APT data: Global mass spectra of **(a)** as-milled NC-(AlCoCrFe)_98.5_Zr_1.5_ and **(b)** NC-(AlCoCrFe)_99_Zr_1_ annealed at 873 K for 4 h; and mass spectra of selected **(c)** grain interior and **(d)** GB of NC-(AlCoCrFe)_99_Zr_1_ annealed at 873 K for 4 h.

Figure S2a and b show the global mass spectra of the APT data for as-milled NC-(AlCoCrFe)_98.5_Zr_1.5_ and NC-(AlCoCrFe)_99_Zr_1_ annealed at 873 K for 4 h, respectively, with identified mass-to-charge-state ratio peaks. All the relevant and major peaks are identified, except a few unindexed peaks that mainly correspond to hydrides. These are a result of the atom probe analysis chamber conditions—residual gaseous hydrogen in the ultra-high vacuum chamber, aided by cryogenic temperature, is adsorbed onto the specimen surface [7]. From the mass spectra, some identified peaks overlapped, making it challenging to deconvolute; this is due to the complexity of the alloy under study. For instance, Cr^3+^ and O^+^ ions overlapped at 16 Da; this contributed to O^+^ dominance in the mass spectrum. The O^+^ may be from the minute traces of oxygen in the samples. Moreover, ZrO^++^ overlapped with Cr^+^ and Fe^+^ at 53, 54, and 55 Da—ZrO^++^ in minute amounts may be influenced by Cr^+^ and Fe^+^ in the mass spectrum for all samples. ZrO^++^ can also be due to the preferential evaporation of O and Zr species [8]—Zr is going to evaporate as both single ions and molecular ZrO species. These are both going to contribute to the final decomposed Zr concentration, represented as a single point in the reconstruction [9]. The local mass spectra for a selected grain interior and GB of annealed NC-(AlCoCrFe)_99_Zr_1_ presented in Figure S2c and d, respectively, are similar to the global mass spectra; this shows the presence of similar elements (Al, Co, Cr, Fe, and Zr) in both the bulk needle and the regions of interest.

**Figure S3**: **(a-c)** 2D HES-XRD pattern of NC-AlCoCrFe: **(a1)** in the as-milled state; **(a2)** after ~2 h 38 min of in-situ annealing at 583 K; **(a3)** after ~7 h 30 min of in-situ annealing at 583 K; **(b1)** after ~30 min of in-situ annealing at 793 K; **(b2)** after ~3 h of in-situ annealing at 793 K; **(b3)** after ~5 h 36 min of in-situ annealing at 793 K; **(c1)** after ~30 min of in-situ annealing at 1003 K; **(c2)** after ~5 h of in-situ annealing at 1003 K; **(c3)** after cooling in air to 423 K, and **(d-f)** 2D HES-XRD pattern of NC-(AlCoCrFe)_98.5_Zr_1.5_: **(d1)** in the as-milled condition; **(d2)** after ~1 h of in-situ annealing at 583 K; **(d3)** after ~5 h 48 min of in-situ annealing at 583 K; **(e1)** after ~30 min of in-situ annealing at 793 K; **(e2)** after ~2 h 24 min of in-situ annealing at 793 K; **(e3)** after ~5 h of in-situ annealing at 793 K; **(f1)** after ~42 min of in-situ annealing at 1003 K; **(f2)** after ~5 h of in-situ annealing at 1003 K; and **(f3)** after cooling in air to 384 K. The grayscale represents the peak intensity of the diffracted X-ray (counts per 0.1 second).

**Figure S4**: Spatially resolved WC coefficients of annealed NC-(AlCoCrFe)_99_Zr_1_ taken from the matrix through to the GBs for each pair of species in the: **(a)** entire ROI 2 of Figure 11c; **(b)** ROI 2 of in Figure 11c taken solely from the matrix; **(c)** ROI 2 of Figure 11c taken solely from the GB; **(d)** entire ROI 3 of Figure 11d; **(e)** ROI 3 of Figure 11d taken solely from the matrix; and **(f)** ROI 3 of in Figure 11d taken solely from the GB. For interpretation of the references to color in this legend, the reader is referred to the web version of this article.

**Figure S5**: Averaged *resolved* WC coefficients of annealed NC-(AlCoCrFe)_99_Zr_1_ taken from the matrix through to the GBs for each pair of species in the: **(a)** entire ROI 2 in *Figure 11c*; **(b)** ROI 2 taken solely from the matrix; **(c)** ROI 2 taken solely from the GB; **(d)** entire ROI 3 in *Figure 11d*; **(e)** ROI 3 taken solely from the matrix; and **(f)** ROI 3 taken solely from the GB.

**Supplementary Video Captions**

**Supplementary video, S1**: Response of NC-AlCoCrFe during in-situ annealing at different temperatures and times in HES-XRD.

**Supplementary video, S2**: Response of NC-(AlCoCrFe)_98.5_Zr_1.5_ during in-situ annealing at different temperatures and times in HES-XRD.

**References**

[1] F. Zhang, Y. Tong, K. Jin, H. Bei, W.J. Weber, Y. Zhang, Lattice distortion and phase stability of Pd-Doped NiCoFeCr solid-solution alloys, Entropy. 20 (2018). https://doi.org/10.3390/e20120900.

[2] M.H. Tsai, J.W. Yeh, High-entropy alloys: A critical review, Mater. Res. Lett. 2 (2014) 107–123. https://doi.org/10.1080/21663831.2014.912690.

[3] M.A. Adaan-Nyiak, I. Alam, E. Jossou, S. Hwang, K. Kisslinger, S.K. Gill, A.A. Tiamiyu, Design and Development of Stable Nanocrystalline High-Entropy Alloy: Coupling Self-Stabilization and Solute Grain Boundary Segregation Effects, Small. 2309631 (2024) 1–18. https://doi.org/10.1002/smll.202309631.

[4] M.A. Adaan-nyiak, I. Alam, G.A. Arcuri, A.A. Tiamiyu, Design of self-stable nanocrystalline high-entropy alloy, Mater. Des. 236 (2023) 112482. https://doi.org/10.1016/j.matdes.2023.112482.

[5] Z. Yu, P.R. Cantwell, Q. Gao, D. Yin, Y. Zhang, N. Zhou, G.S. Rohrer, M. Widom, J. Luo, M.P. Harmer, Segregation-induced ordered superstructures at general grain boundaries in a nickel-bismuth alloy, Science (80-. ). 358 (2017) 97–101. https://doi.org/10.1126/science.aam8256.

[6] C.L. Platt, K.W. Wierman, E.B. Svedberg, R. Van De Veerdonk, J.K. Howard, A.G. Roy, D.E. Laughlin, L-1 0 ordering and microstructure of FePt thin films with Cu, Ag, Au additive, J. Appl. Phys. 92 (2002) 6104–6109. https://doi.org/10.1063/1.1516870.

[7] S.M. Reddy, D.W. Saxey, W.D.A. Rickard, D. Fougerouse, S.D. Montalvo, R. Verberne, A. van Riessen, Atom Probe Tomography: Development and Application to the Geosciences, Geostand. Geoanalytical Res. 44 (2020) 5–50. https://doi.org/10.1111/ggr.12313.

[8] D.W. Saxey, Correlated ion analysis and the interpretation of atom probe mass spectra, Ultramicroscopy. 111 (2011) 473–479. https://doi.org/10.1016/j.ultramic.2010.11.021.

[9] A. Breen, M.P. Moody, B. Gault, A. V. Ceguerra, K.Y. Xie, S. Du, S.P. Ringer, Spatial decomposition of molecular ions within 3D atom probe reconstructions, Ultramicroscopy. 132 (2013) 92–99. https://doi.org/10.1016/j.ultramic.2013.02.014.
